# *Mycobacterium marinum* Degrades Both Triacylglycerols and Phospholipids from Its *Dictyostelium* Host to Synthesise Its Own Triacylglycerols and Generate Lipid Inclusions

**DOI:** 10.1371/journal.ppat.1006095

**Published:** 2017-01-19

**Authors:** Caroline Barisch, Thierry Soldati

**Affiliations:** Department of Biochemistry, Science II, University of Geneva, 30 quai Ernest-Ansermet, Geneva, Switzerland; University of Massachusetts Medical School, UNITED STATES

## Abstract

During a tuberculosis infection and inside lipid-laden foamy macrophages, fatty acids (FAs) and sterols are the major energy and carbon source for *Mycobacterium tuberculosis*. Mycobacteria can be found both inside a vacuole and the cytosol, but how this impacts their access to lipids is not well appreciated. Lipid droplets (LDs) store FAs in form of triacylglycerols (TAGs) and are energy reservoirs of prokaryotes and eukaryotes. Using the *Dictyostelium discoideum*/*Mycobacterium marinum* infection model we showed that *M*. *marinum* accesses host LDs to build up its own intracytosolic lipid inclusions (ILIs). Here, we show that host LDs aggregate at regions of the bacteria that become exposed to the cytosol, and appear to coalesce on their hydrophobic surface leading to a transfer of diacylglycerol O-acyltransferase 2 (Dgat2)-GFP onto the bacteria. *Dictyostelium* knockout mutants for both Dgat enzymes are unable to generate LDs. Instead, the excess of exogenous FAs is esterified predominantly into phospholipids, inducing uncontrolled proliferation of the endoplasmic reticulum (ER). Strikingly, in absence of host LDs, *M*. *marinum* alternatively exploits these phospholipids, resulting in rapid reversal of ER-proliferation. In addition, the bacteria are unable to restrict their acquisition of lipids from the *dgat1&2* double knockout leading to vast accumulation of ILIs. Recent data indicate that the presence of ILIs is one of the characteristics of dormant mycobacteria. During *Dictyostelium* infection, ILI formation in *M*. *marinum* is not accompanied by a significant change in intracellular growth and a reduction in metabolic activity, thus providing evidence that storage of neutral lipids does not necessarily induce dormancy.

## Author Summary

*Mycobacterium tuberculosis* (*Mtb*) survives the human immune defence mechanisms leading to latent tuberculosis in one third of the world population. The ability to persist latently in human macrophages is due to a remarkable physiological change that is accompanied by a slowdown in replication, low metabolism, and phenotypic tolerance to antibiotics. It was proposed that fatty acids released from bacterial intracytosolic lipid inclusions (ILIs), a characteristic of dormant *Mtb*, serve as carbon source during reactivation from dormancy. We show here that bacteria accumulate ILIs even in a *Dictyostelium* mutant that is deficient in triacylglycerol synthesis and therefore incapable to build up lipid droplets. In addition, the accumulation of ILIs is not sufficient to induce a dormancy-like phenotype in *M*. *marinum* inside its host *Dictyostelium*. Moreover, we propose an alternative lipid transfer route from the host to the pathogen via degradation and recycling of host phospholipids.

## Introduction

Tuberculosis (Tb) is caused by *Mycobacterium tuberculosis* (*Mtb*) and remains one of the most deadly infectious diseases in the world. In immunocompetent people, *Mtb* is contained by host defence mechanisms, resulting in 2–3 billion people carrying latent Tb without clinical disease. During latency, the bacteria transition to dormancy, defined here as a slowly- or non-replicating state that is characterized by low metabolic activity and resistance to antibiotic treatment. However, when the immune system of the host is weakened, e.g. in diabetes or upon HIV infection, dormant bacteria can be reactivated leading to active infectious Tb (reviewed in [1]).

One of the hallmarks of Tb is the formation of granulomas, i.e. compact and organized structures comprised of immune cells, such as blood-derived macrophages, foamy macrophages, epithelioid cells and multinucleated giant cells surrounded by a ring of lymphocytes (reviewed in [2]). Lipid droplet (LD) accumulation in macrophages, which leads to their “foamy” appearance, is another major characteristic of Tb, but also of other mycobacteria infections caused by *M*. *avium* [3], *M*. *bovis* [4] and *M*. *leprae* [5], and was not observed upon infection with non-pathogenic species, such as *M*. *smegmatis* [3]. Intracellular mycobacteria have been seen in the vicinity of LDs [3, 6] and are able to access these lipid stores to acquire fatty acids (FAs; [7]) to build up their own intracytosolic lipid inclusions (ILIs) for times of nutrient deprivation [6]. Interestingly, the formation of ILIs is a main characteristic of dormant bacteria [7, 8] and with the help of an *in vitro* dormancy model it was proposed that FAs, released form bacterial TAGs, serve as carbon source to initiate replicative activities [9].

We have established *Dictyostelium discoideum* (hereafter referred to as *Dictyostelium*) as a model for foamy macrophages in mycobacterial infections. Instead of *Mtb*, we infect *Dictyostelium* with *Mycobacterium marinum*, a natural pathogen of poikilotherms, and the closest relative to the *Mtb* complex [10]. Since virulence genes are highly conserved between both species and the infections share a high level of similarity at the cellular level, *M*. *marinum* is a widely accepted model for Tb (reviewed in [11]). This is strengthened by the observation that *M*. *marinum* also accesses host lipids, by re-directing LDs to the mycobacterium-containing vacuole (MCV), leading to an accumulation of neutral lipids inside the compartment [12]. In addition, *M*. *marinum* was recently shown to enter a dormant-like state in zebrafish with limited mortality rates and stable bacteria loads [13]. Interestingly, mycobacteria growth was restored when the immune system of the fish was weakened by γ-irradiation. Whether lipid metabolism or LDs play a role during dormancy of *M*. *marinum* is so far poorly understood.

LDs store energy in the form of neutral lipids, such as triacylglycerols (TAGs) and sterol esters (SEs), and are present in virtually every cell type ranging from simple organisms such as bacteria and amoebae to plants and mammals. TAGs are synthesised in at least two different ways, but commonly the last step, the acylation of diacylglycerol (DAG) that leads to the formation of TAG, is catalysed by diacylglycerol O-acyltransferases (Dgats; (reviewed in [14]).

Mammalian cells express two Dgat enzymes belonging to two proteins families with limited sequence similarity. Dgat1 is localized at the membrane of the endoplasmic reticulum (ER) where it mainly uses FAs released from nutrients or by lipolysis of LDs for TAG synthesis. Dgat2 is at the ER, mitochondria-associated membranes, and surface of LDs [15] and prefers newly synthesized FAs [16]. Dgat2 was shown to be the major Dgat enzyme in mammals, since mice lacking Dgat2 are not viable and die soon after birth [17]. Interestingly, in adipocytes differentiated from murine embryo fibroblasts (MEFs) derived from *dgat1&2* double knockout (DKO) animals, no LDs were formed at all. In contrast, in *dgat1* and *dgat2* single knockout (KO) MEFs lipid accumulation was observed, even in absence of exogenous FAs during differentiation [18]. In macrophages deficient for both Dgats, no LDs were observed after oleic acid addition, whereas LDs containing SEs accumulated after addition of acetylated low density lipoproteins [18]. In *Dictyostelium*, two Dgat proteins were described recently [19]. As in mammalian cells, Dgat1 is located at the ER, while Dgat2 was observed exclusively on LDs. Interestingly, *dgat2*-deficient cells produce the same amount of TAGs as wild type cells [19]. Surprisingly, Dgat1 has more prominent activity in *Dictyostelium*, since *dgat1* KO and *dgat1&2* DKO cells were dramatically affected in their capacity to form TAGs after incubation with exogenous FAs [19].

The role of host Dgat enzymes in bacterial or viral infections is poorly understood. Recently, Dgat1 activity was shown to be important for hepatitis C particle formation, but not relevant for viral replication and translation [20]. Dgat proteins also have a function in *Chlamydia trachomatis* infection. When MEFs derived from *dgat1&2* DKO animals were infected with *C*. *trachomatis*, less infectious progeny was generated than in wild type MEFs, leading the authors to conclude that host TAGs are required for chlamydial replication [21].

Here, we first decipher the role of *Dictyostelium* Dgat proteins during *M*. *marinum* infection and bring evidence that intracellular mycobacteria are not only able to acquire FAs from TAGs stored in host LDs, but also from host phospholipids. In addition, in host mutants unable to generate LDs, *M*. *marinum* appears to accumulate ILIs in an unrestricted manner without significant impact on its proliferation.

## Results

### Dgat2-GFP-labelled LDs aggregate around cytosolic bacteria

**Fig 1:**
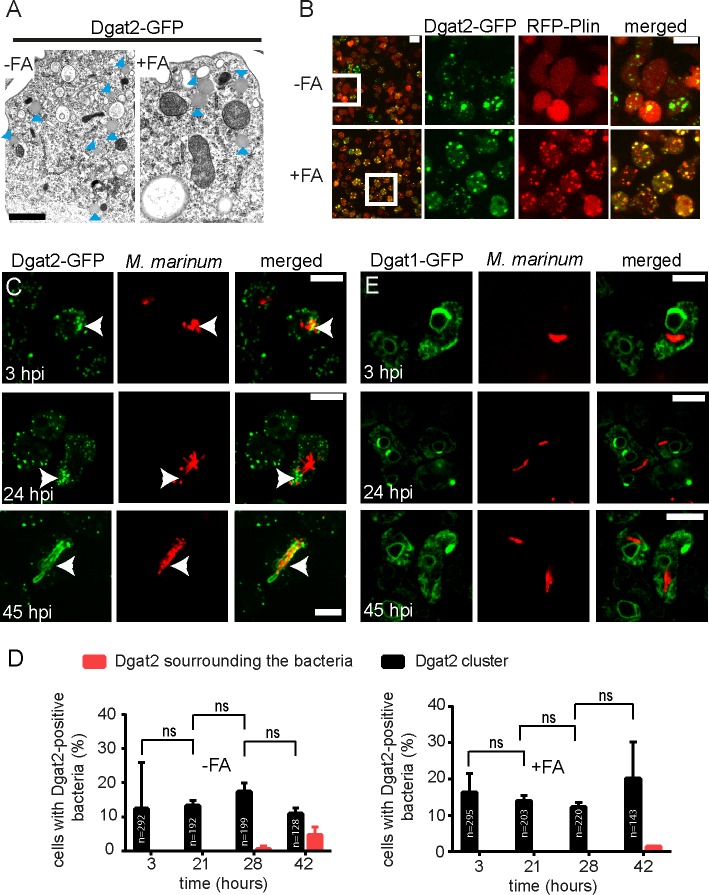
Localization of Dgat1- and Dgat2-GFP during infection of *Dictyostelium* with *M*. *marinum*. A. LDs with their typical morphology are formed in cells overexpressing Dgat2-GFP even without FA supplementation. Cells that were treated with and without FAs were fixed and processed for EM. Arrowheads label LDs. Scale bar, 1 μm. B.Dynamics of RFP-Plin and Dgat2-GFP in *Dictyostelium* treated with exogenous FAs. In axenic medium without FA supplementation, RFP-Plin is cytosolic whereas Dgat2-GFP is located on LDs. Upon treatment with exogenous FAs, RFP-Plin translocates to the surface of LDs where it co-localizes with Dgat2-GFP. *Dictyostelium* expressing both RFP-Plin and Dgat2-GFP was cultured in medium supplemented with FAs and a time-lapse movie was recorded with 5 min frame intervals. Shown is the maximum z-projection of 6 sections spaced 1.5 μm apart. Scale bars, 10 μm. C. Dgat2-GFP-positive LDs cluster at bacterial poles. *Dictyostelium* expressing Dgat2-GFP was infected with mCherry-expressing mycobacteria. Samples for live imaging were taken at 3, 24 and 45 hpi. *Dictyostelium* was fed with FA prior to infection. Arrows point to LD clusters. Scale bars, 10 μm. D. Quantification of C. The number of *Dictyostelium* cells harbouring bacteria that co-localize with LDs aggregates was stable over the time course of infection. Bacteria surrounded by Dgat2-GFP were only observed at late stages, as judged by quantification using z-projections. The statistical significance was calculated with an unpaired t-test (* p<0.05, ** p<0.01). Bars represent the mean and SD of two independent experiments. E. Dgat1-GFP is enriched at the ER and at the perinuclear ER during infection of *Dictyostelium* with mCherry-expressing *M*. *marinum*. Samples for live imaging were taken at 3, 24 and 45 hpi. *Dictyostelium* was fed with FA prior to infection. Scale bars, 10 μm.

Previously, we have shown that *M*. *marinum* uses host-derived lipids to build up more and larger ILIs during the infection of *Dictyostelium* [12]. Consequently, we wondered whether host proteins with a function in TAG synthesis, such as Dgat1 and Dgat2, were involved in that process. Importantly, the localization and the function of both Dgat proteins in *Dictyostelium* was thoroughly assessed in a previous study [19]. The authors showed that overexpression of Dgat2-GFP induces TAG production and LD generation in *Dictyostelium* without FA supplementation, probably by rerouting metabolic pathways [19]. In addition, it was observed that Dgat2-GFP overexpression rescues the capacity of the *dgat1&2* DKO to synthesise TAGs [19], leading to the generation of LDs with an ultrastructural morphology indistinguishable from LDs induced by FA supplementation (Fig 1A). Since Plin, the *Dictyostelium* homologue of Perilipin, is cytosolic in the absence of FAs and relocates to the LD surface when the medium is supplemented with FAs, its localization was monitored in a strain expressing Dgat2-GFP. Strikingly, Plin was cytosolic under standard conditions, even though LDs decorated with Dgat2-GFP were observed. Soon after FA addition, Plin then relocated to LDs, where both proteins co-localized (Fig 1B; S1 Movie). Together with the observation that Dgat2-GFP-labelled LDs are LD540 positive [19], this result suggests the presence of two different LD types in *Dictyostelium* that are either artificially induced by overexpressing Dgat2-GFP, or are generated by the different functions of Dgat2 and Plin in TAG synthesis and lipolysis, respectively.

When the localization of both Dgat proteins was monitored during infection, we observed that many of the Dgat2-GFP-positive LDs clustered around the MCV (Fig 1C). While this clustering of LDs was observed at all stages, labelling of the entire bacteria was rarely seen and occurred only at late infection stages (Fig 1C and 1D; black and red bars, respectively). The localization of Dgat1-GFP did not change during infection, remaining at the ER at all stages (Fig 1E). Addition of exogenous FAs, which leads to further induction of LDs did not influence the localization of Dgat1-GFP and Dgat2-GFP, respectively (Fig 1D).

**Fig 2:**
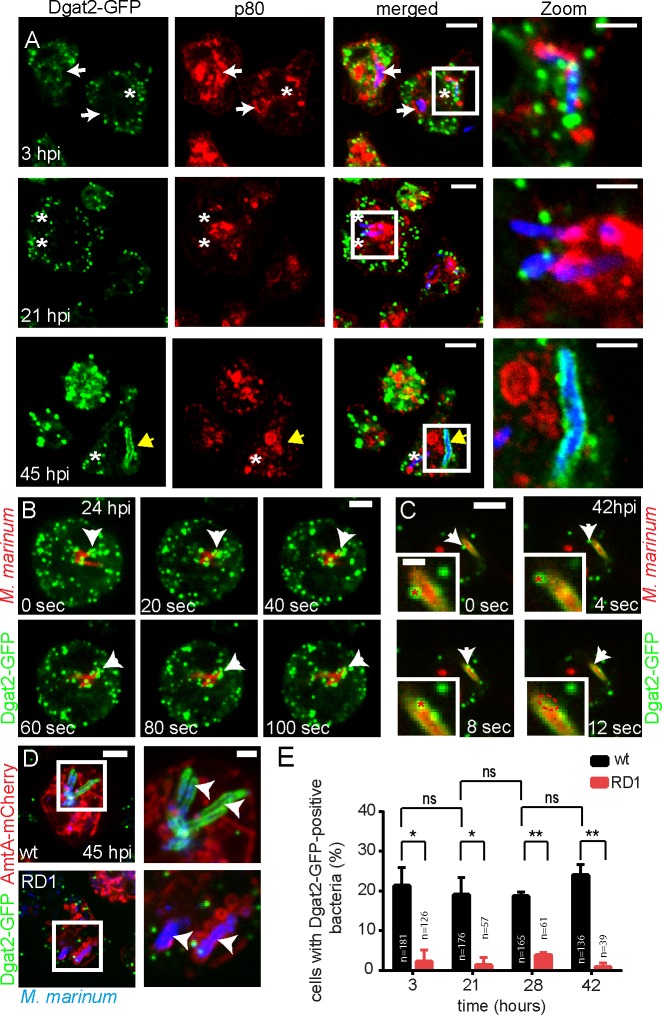
Dynamics of Dgat2-GFP-LDs during infection. A. Dgat2-GFP-positive LDs attach to the bacteria when they are exposed to the cytosol (3 and 21 hpi). At a later infection stage, Dgat2-GFP completely surrounds a cytosolic bacterium (45 hpi). White arrows point to bacteria that are inside the p80-positive MCV. Asterisks label bacteria that are partially exposed to the cytosol. The yellow arrow points to a cytosolic bacterium that is completely surrounded by Dgat2-GFP. Scale bars, 10 μm; Zoom, 2 μm. B. Dgat2-GFP-labelled LDs stick to an intracellular mCherry-expressing mycobacterium. A time-lapse movie was recorded at 24 hpi with 5 sec frame intervals. Arrows point to LDs aggregated at the surface of the bacterium. Scale bar, 3 μm. C. Coalescence of a Dgat2-GFP-positive LD with an mCherry-expressing mycobacterium surrounded by Dgat2-GFP (arrows). A time-lapse movie was recorded at 42 hpi with 2 sec frame intervals. Arrows point to an LD that coalescences onto the bacterium. Asterisks label the same LD in the Zoom. Scale bar, 5 μm; Zoom, 2μm. D. Dgat2-GFP surrounds a cytosolic wild type *M*. *marinum* negative for AmtA-mCherry. No co-localization was observed with *M*. *marinum* ΔRD1. Arrows label intracellular bacteria. Samples were taken at 45 hpi and bacteria stained with Vybrant Ruby. Scale bars, 5 μm. E. Quantification of D. While clusters of Dgat2-GFP-labelled LDs were frequently observed close to wild type *M*. *marinum*, only a few LDs associated with the RD1 mutant. Dgat2-GFP-positive bacteria were counted in maximum z-projections. Statistical significance was calculated with an unpaired t-test (* p<0.05, ** p<0.01). Bars represent the mean and SD of two independent experiments. For all the experiments presented in Fig 2 *Dictyostelium* was fed with FA prior to infection.

To assess whether the Dgat2-GFP-labelled bacteria were inside a vacuole or cytosolic, infected Dgat2-GFP-expressing cells were PFA-fixed at 3, 21 and 45 hpi and stained for the endosomal protein p80, a predicted copper transporter enriched at the MCV [22]. At 3 and 21 hpi, LDs converged to regions devoid of p80, and thus cytosolically exposed (Fig 2A, asterisks). At late infection stages (45 hpi), Dgat2-GFP surrounded bacteria that had fully escaped from the MCV to the cytosol (Fig 2A, yellow arrow). In contrast to the *Dictyostelium* homologue of perilipin, which is attracted to all the bacteria as soon as their surface is exposed to the cytosol [12] the homogenous labelling of Dgat2-GFP around cytosolic bacteria, was only observed late and for a small fraction of cytosolic bacteria (Fig 1D). In addition, we observed by time-lapse microscopy that, as soon as *M*. *marinum* initiated its escape from the vacuole to the cytosol at around 24 hpi, the Dgat2-GFP-positive LDs clustered at the emerging bacterial poles, and appeared somehow attached to the bacterial surface, as judged by their coordinated movement (Fig 2B, arrows; S2 Movie). Remarkably, the LDs subsequently seemed to coalesce with the bacterial surface leading to a homogenous circum-bacteria labelling (Fig 2C, arrows; S3 Movie).

Interestingly, clusters of Dgat2-GFP-LDs around bacteria were rarely seen when *Dictyostelium* was infected with ΔRD1 mutant bacteria (Fig 2D and 2E), which escape five- to ten-fold less efficiently from the MCV than wild type *M*. *marinum* [23]. Moreover, complete labelling of the bacteria by Dgat2-GFP never occurred in infections with ΔRD1 mutant bacteria (Fig 2D).

In summary, we observed that Dgat2-GFP-positive LDs first cluster around bacterial poles that become exposed to the cytosol, and finally coalesce with the surface of cytosolic bacteria.

### Perilipin restricts Dgat2 from the surface of cytosolic bacteria

Since Dgat2 and Plin have been both observed at the surface of cytosolic *M*. *marinum*, the dynamics of both proteins during infection was monitored in infected *Dictyostelium* expressing RFP-Plin and Dgat2-GFP. At 45 hpi, a sample was transferred into a μ-dish, and the unlabelled bacteria were visualised by the DNA-dye Vybrant Ruby. Interestingly, we observed that bacteria strongly labelled by Dgat2-GFP (white arrows) were only weakly surrounded by RFP-Plin (yellow arrow) and vice versa (S1A Fig). Since RFP-Plin was located to the majority of bacteria and Dgat2-GFP to only a minor fraction (S1B Fig), we suggest that Plin restricts Dgat2 from cytosolic bacteria, in a way reminiscent of protein crowding around LDs, as recently reported [24].

### Bacteria accumulate ILIs in the *dgat1&2* DKO

**Fig 3:**
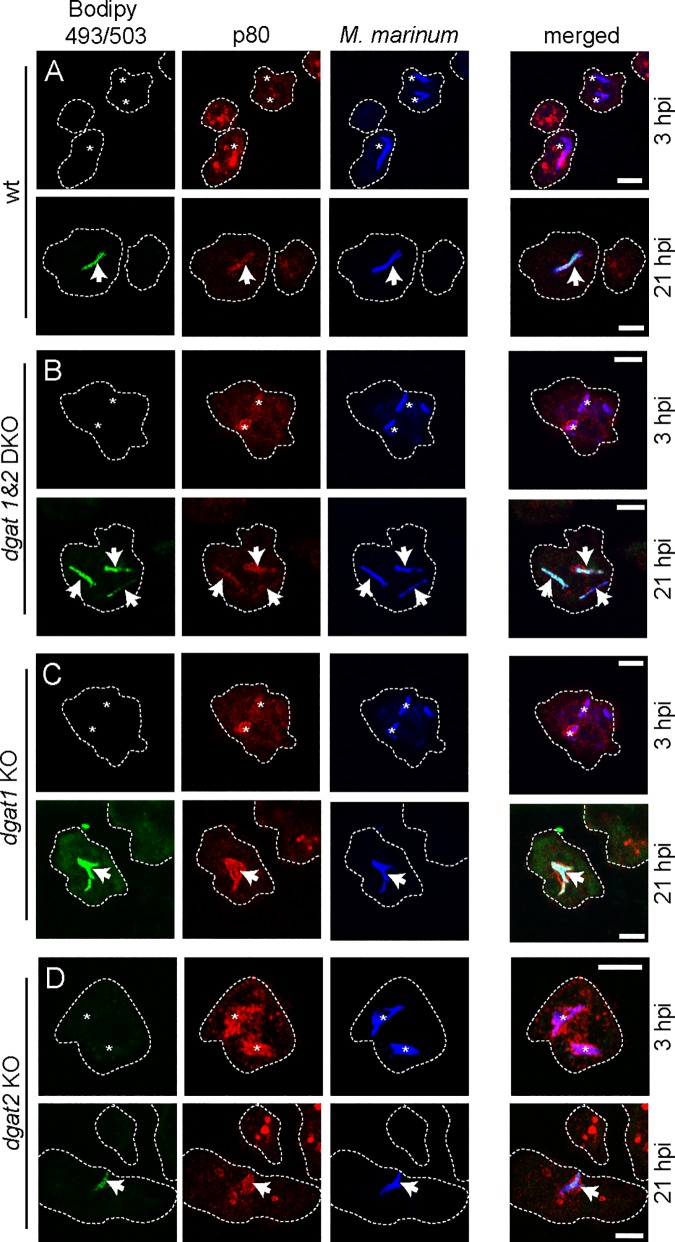
Bacteria accumulate ILIs in the *dgat* KO mutants. Cells of (A) wild type, (B) *dgat1&2* DKO, (C) *dgat1* KO and (D) *dgat2* KO and were infected with mCherry-expressing *M*. *marinum*. At 3 hpi bacteria are lean (asterisks) whereas at 21 hpi bacteria harbour many ILIs in all cell types (arrows). Cells were fed with FAs prior to infection. At the indicated time points samples were fixed with PFA/picric acid, and MCVs visualized by staining for p80. Bacterial ILIs were stained with Bodipy 493/503. Scale bar, 5 μm.

In wild type *Dictyostelium*, LDs cluster around the MCV minutes after uptake, probably serving as a source of FAs for the invading bacteria to build up their ILIs. *Dictyostelium* cells lacking both Dgat enzymes synthesize extremely low levels of TAGs [19]. Accordingly, we wondered whether the intracellular bacteria are still able to produce ILIs in cells deficient for one or both Dgats. To monitor bacterial ILIs, wild type or *dgat* mutants were infected with mCherry-expressing *M*. *marinum* and fixed with paraformaldehyde (PFA) at 3 and 21 hpi, respectively. ILIs were stained and the MCVs labelled for p80. Importantly, cells were fed with exogenous FAs before infection. In both wild type and *dgat1&2* DKO cells, the bacteria harboured many more and larger ILIs at 21 hpi than at 3 hpi (Fig 3A and 3B), but, at both time points, most bacteria that accumulated ILIs were still inside their MCV (Fig 3A–3D). In addition, intracellular bacteria did also generate ILIs in the *dgat1* and *dgat2* KO cells (Fig 3C and 3D). Of note, when *Dictyostelium* cells are fixed with PFA and permeabilised with the detergent TritonX-100, the LD-lipids are extracted, affecting the usual LD staining. Importantly, probably due to their waxy cell wall, lipids of the bacteria are not drastically affected by TritonX-100 and are stained by common LD dyes.

**Fig 4:**
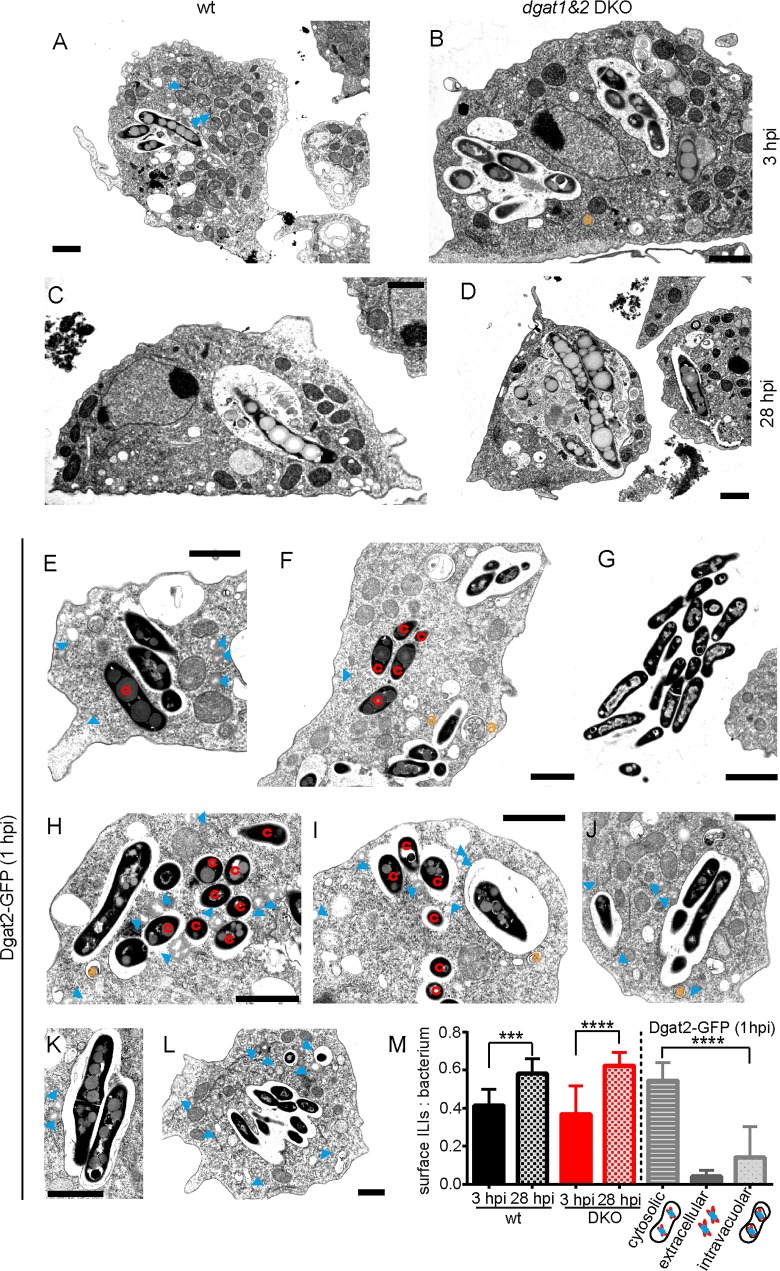
LD and ILI dynamics during infection. A.—D. Bacteria accumulate ILIs in the wild type and the *dgat1&2* DKO. E. and F. Cytosolic bacteria harbour more ILIs than bacteria inside an MCV. G. Extracellular bacteria are lean. H. and I. LDs translocate to cytosolic bacteria J.—L. LDs are recruited to the vicinity of the MCV early in infection. *Dictyostelium* wild type (A and C) and *dgat1&2* DKO cells (B and D) or cells expressing Dgat2-GFP (E-L) were infected with unlabelled *M*. *marinum* wild type. At the indicated time points, samples were fixed and further processed for EM. Arrowheads label LDs. a: autophagosomes, c: cytosolic bacteria. Scale bars, 1 μm. M. Quantification of the ILI surface per bacterium as a fraction of the total bacterium surface. For each condition ILIs of 10 to 13 bacteria were quantified using FIJI. The statistical significance was calculated with an unpaired t-test (*** p<0.001, **** p<0.0001). For all the experiments presented in Fig 4 *Dictyostelium* was fed with FA prior to infection.

When wild type and *dgat1&2* DKO were infected with *M*. *marinum*, fixed and processed for EM (Fig 4A–4D), again, bacteria showed accumulation of many more ILIs at 28 hpi (Fig 4C and 4D) compared to 3 hpi (Fig 4A and 4B). Besides, no obvious differences were observed between the infected wild type and *dgat1&2* DKO cells, neither in the morphology of the MCV, nor of the bacteria.

In conclusion, despite the incapacity of *dgat1* and *dgat1&2* mutants to synthesise TAGs, a large amount of ILIs accumulated at later infection stages.

### LDs translocate to the vicinity of the MCV and to cytosolic *M*. *marinum*

In *Dictyostelium*, around 10–20% of infected bacteria are observed in the cytosol at any time between 1 and 25 hpi, whereas the major wave of escape from the compartment is observed between 24 and 50 hpi, as confirmed by Plin-labelling [12]. In *Dictyostelium* cells overexpressing Dgat2-GFP, we also observed ILI accumulation in the few bacteria that escaped to the cytosol early in infection (Fig 4E and 4F). In contrast, extracellular bacteria (Fig 4G) and vacuolar bacteria (Fig 4E and 4F, Fig 4H–4L) accumulated less ILIs at 1 hpi. Interestingly, numerous host LDs were observed in the vicinity of the cytosolic bacteria (Fig 4H and 4I), confirming our previous results (Figs 1C and 2A–2C) and leading us to conclude that *M*. *marinum* is able to access lipids from Dgat2-GFP-labelled LDs to build up its own stores. Finally, in *Dictyostelium* cells overexpressing Dgat2-GFP, we also observed LD clustering around the MCV at 1 hpi (Fig 4J–4L), confirming our previous findings [12]. Importantly, quantification of total ILI surface per bacterium was performed and confirmed the observations described above (Fig 4M).

### ER-membrane proliferation in *dgat1&2* cells

**Fig 5:**
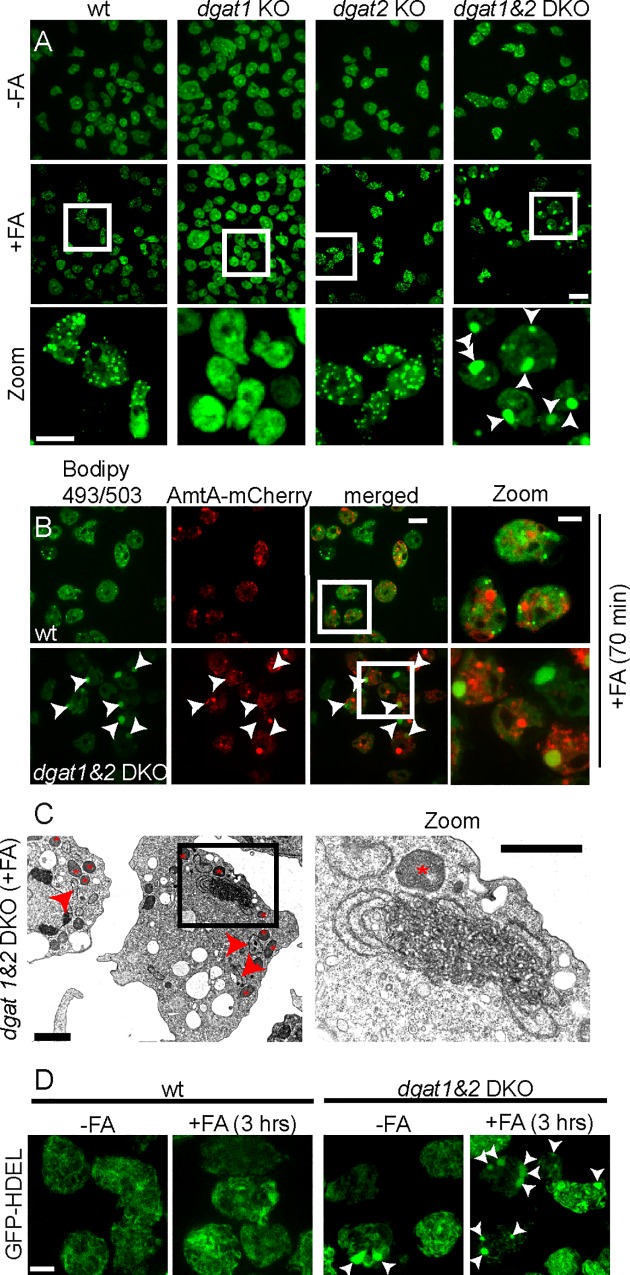
Excess FAs leads to ER-membrane proliferation in *dgat1&2* DKO cells. A. LDs are formed in wild type and *dgat2*, but not in *dgat1* KO cells. Instead of LDs, massive Bodipy-positive structures were observed in the *dgat1&*2 DKO (arrowheads). FAs were added to the culture medium and a time-lapse movie was recorded with 10 minute frame intervals. Shown are maximum z-projections of 6 sections 1.5 μm apart taken after 180 min. Scale bars, 10 μm. B. The neutral lipid structures in the *dgat1&2* DKO (arrowheads) are not of endosomal nature. Wild type or *dgat1&2* DKO cells expressing AmtA-mCherry were incubated with FAs and a time-lapse movie with 5 min frame intervals was recorded. Shown is a representative image taken after 70 min. Scale bar, 10 μm; Zoom 5 μm. C. The neutral lipid structures in the *dgat1&2* DKO are formed by ER-membranes. *Dictyostelium* was fed 3 hours with FAs before fixation with glutaraldehyde. Asterisks label mitochondria that have been seen close to the ER-membrane-proliferations. Arrowheads point to long ER-strands. D. GFP-HDEL accumulates in the ER-membrane proliferations in the *dgat1&2* DKO. Images of *Dictyostelium* expressing GFP-HDEL were taken under normal conditions (-FAs) and after 3 hrs incubation with FAs (+FAs). Shown are maximum z-projections. Arrowheads point to ER-membrane proliferations. Scale bar, 5 μm.

Since *Dictyostelium* cells deficient in *dgat1* and *dgat1&2* synthesise extremely low levels of TAGs [19], we wondered in which form the exogenous FAs were stored, and thus monitored by time-lapse microscopy the fate of lipids in various *dgat* mutants (Fig 5A). While Bodipy 493/503-labelled LDs were generated in wild type and *dgat2* mutant cells, as expected no LDs were visible in *dgat1-*deficient cells (Fig 5A). Strikingly, in the *dgat1&2* DKO, Bodipy 493/504 revealed intensely stained neutral lipid structures (Fig 5A, arrowheads), which became even larger upon FAs supplementation (S4 Movie). Importantly, incubation with exogenous FAs did not affect the viability of wild type and *dgat1&2* DKO cells (S2 Fig).

In NPC disease and other lysosomal storage diseases, excessive lipids accumulate in lysosomes [25]. In order to visualise the endo-lysosomal membrane system, a fusion of mCherry with AmtA, an ammonium transporter [26, 27] that localises to all endosomes and phagosomes in *Dictyostelium* [26, 27], was expressed in *dgat1&2* deficient cells. Interestingly, after FA supplementation no significant co-localization was observed between AmtA-mCherry and the neutral lipid structures (Fig 5B, arrowheads), implying that they are not of endosomal nature.

To test for morphological aberrations, we fixed wild type and cells deficient in *dgat1*, *dgat2* and *dgat1&2* after feeding with FAs (S3A–S3D Fig). Whereas LDs were observed in wild type and the *dgat2* KO cells (S3A and S3C Fig, blue triangles), LDs were completely absent from the *dgat1* and *dgat1&2* deficient cell lines (S3B and S3D Fig). In contrast to wild type and *dgat2* KO cells, where the rough ER morphology was normal, massive proliferation of ribosome-studded ER membranes became visible in the *dgat1&2* DKO (Figs 5C and S3D; red arrows). An intermediate phenotype was apparent in *dgat1* cells (S3B Fig). In addition, mitochondria were observed in close vicinity of the elongated ER-membrane structures in *dgat1* and d*gat1&2* deficient cells (Figs 5C, S3B and S3D; red asterisks). In *Dictyostelium*, the HDEL motif acts as an ER-retrieval signal [28, 29]. Accordingly, in contrast to wild type cells, ER-membrane proliferation was highlighted in *d*g*at1&2* DKO cells expressing GFP-HDEL (Fig 5D, arrowheads). At the same time, these proliferations were stained by BodipyC12 (S3E Fig). Importantly, ER-proliferations were more frequent and more massive, when *dgat1&2* DKO cells were fed with FAs.

In summary, *Dictyostelium* cells deficient in both *dgat* genes do not form LDs, but channel the excess of FAs into phospholipids that build massive ER-membrane proliferations, which are in close contact with mitochondria and can be visualized by live microscopy in cells expressing GFP-HDEL.

### ER-membrane proliferations are rapidly depleted from infected cells

**Fig 6:**
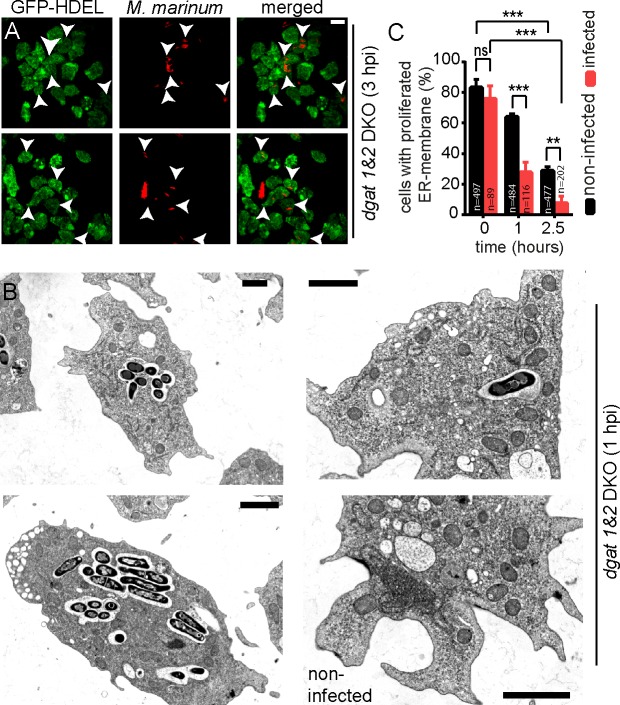
ER-membrane proliferations are depleted in infected cells. A. *Dgat1&2* DKO cells expressing GFP-HDEL were infected with mCherry-expressing mycobacteria. Shown are two maximum z-projections taken at 3 hpi. Scale bar, 10 μm. B. The *dgat1&2* DKO was infected with unlabelled *M*. *marinum*. Cells were fixed at 1 hpi and further processed for EM. Shown are three examples of infected cells, and one of an uninfected *Dictyostelium* cell. Scale bars, 2 μm. C. The percentage of cells showing proliferation of ER membranes as a function of time. *Dgat1&2* DKO cells expressing GFP-HDEL were either infected with mCherry-expressing *M*. *marinum* or left non-infected. The statistical significance was calculated with an unpaired t-test (** p<0.01, *** p<0.001). Bars represent the mean and SD of three independent experiments. At the indicated time points, images were taken for manual quantification. For all the experiments presented in Fig 6 *Dictyostelium* was fed with FA prior to infection.

When *dgat1&2* DKO cells expressing GFP-HDEL were infected with *M*. *marinum*, no significant and direct contact was noticed between the ER-proliferations and the MCV or with the cytosolic bacteria (Fig 6A). Interestingly, the excessive ER structures disappeared from most infected cells, even at very early stages of infection (Fig 6A, arrows). When *dgat1&2* DKO cells were fixed and processed for EM, again, no specific accumulation of ER structures was visible in direct vicinity of the MCV and cytosolic bacteria (Fig 6B). The ER-proliferations remained clearly visible in neighbouring non-infected cells (Fig 6B). Quantification of these observations confirmed that the ER-proliferations disappeared faster from infected than from non-infected cells (Fig 6C).

### *M*. *marinum* uses host phospholipids to build up ILIs

Despite the deficiency of the *dgat1&2* DKO to synthesise TAGs [19] and LDs (Fig 5A), we observed that *M*. *marinum* accumulated many ILIs in the *dgat1&2* DKO during infection (Figs 3 and 4D). Instead of synthesising TAGs, *Dictyostelium* shuttles exogenous FAs into host phospholipids leading to ER-membrane proliferations in the *dgat1&2* DKO (Fig 5C). The rapid disappearance of the ER-proliferations early in infection led us to conclude that *M*. *marinum* somehow accesses host phospholipids.

To specifically label the phospholipids of *Dictyostelium* prior to infection, we incubated wild type and *dgat1&2* DKO cells overnight with exogenous FAs to induce lipid synthesis and storage and chased afterwards for 1 hour with Topfluor-Lysophosphatidylcholine (PC) complexed to defatted Bovine Serum Albumin (BSA). Topfluor-LysoPC is a ubiquitous lipid that is amongst others generated by hydrolysis of PC by phospholipase (PL) A2. In addition, we decided to use Topfluor-LysoPC, because it is much more soluble than PC, and is taken up easily by *Dictyostelium*. We expected that Topfluor-LysoPC becomes acylated, probably by the action of lysophospholipid acyltransferase at the ER, giving rise to Topfluor-PC that should be present in almost all cellular membranes in analogy to PC [30]. Moreover, the fluorescent label of Topfluor-LysoPC is linked to the acyl-chain and not the phosphate group, which should allow us to follow the localization of Topfluor-labelled FAs that are released from Topfluor-LysoPC by host and bacterial enzymes such as phospholipases C.

Interestingly, when non-infected wild type *Dictyostelium* cells were incubated with Topfluor-LysoPC, the label was enriched in the perinuclear ER (asterisks) and in vesicles reminiscent of endosomes in *Dictyostelium* (S4A Fig). In the *dgat1&2* DKO, we observed the same staining of the perinuclear ER (asterisks), but also the proliferation of the ER-membrane (arrowheads) and to a minor extent vesicles were labelled (S4B Fig).

**Fig 7:**
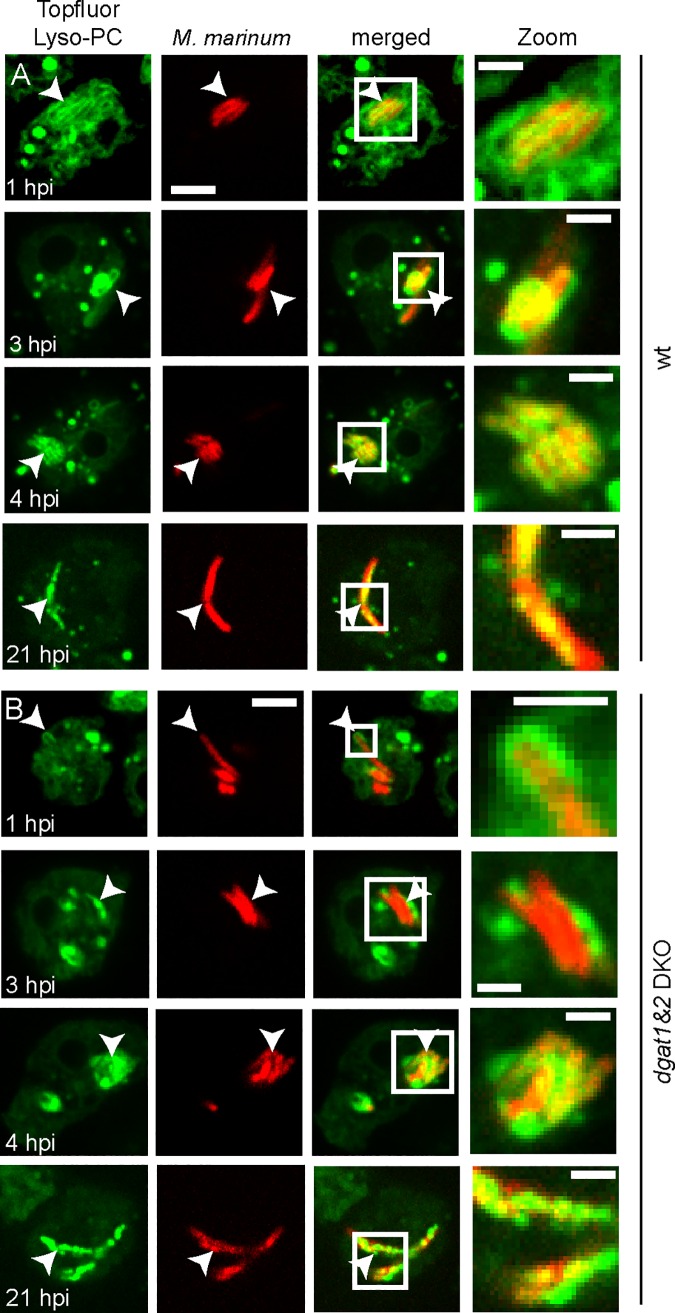
Lipids derived from host phospholipids are transferred to the MCV. A. and B. Topfluor-LysoPC-tagged host lipids first label the membrane of the MCV, accumulate inside the compartment and are finally found inside the bacteria. Phospholipids of wild type (A) and *dgat1&2* DKO (B) were labelled with Topfluor-LysoPC as described in materials and methods. Cells were infected with mCherry-expressing mycobacteria. Images were taken at the indicated time points. Scale bar, 5 μm; Zoom, 2μm.

When Topfluor-LysoPC-labelled cells were infected with *M*. *marinum* (Fig 7), the labelling became first visible at the membrane of the MCV (1–3 hpi), then accumulated inside the compartment (starting from 4 hpi) and finally was integrated into the bacteria themselves (21 hpi). Interestingly, these observations were made both in infected wild type and *dgat1&2* DKO cells, indicating that the same route of lipid transfer is active in both cell lines.

**Fig 8:**
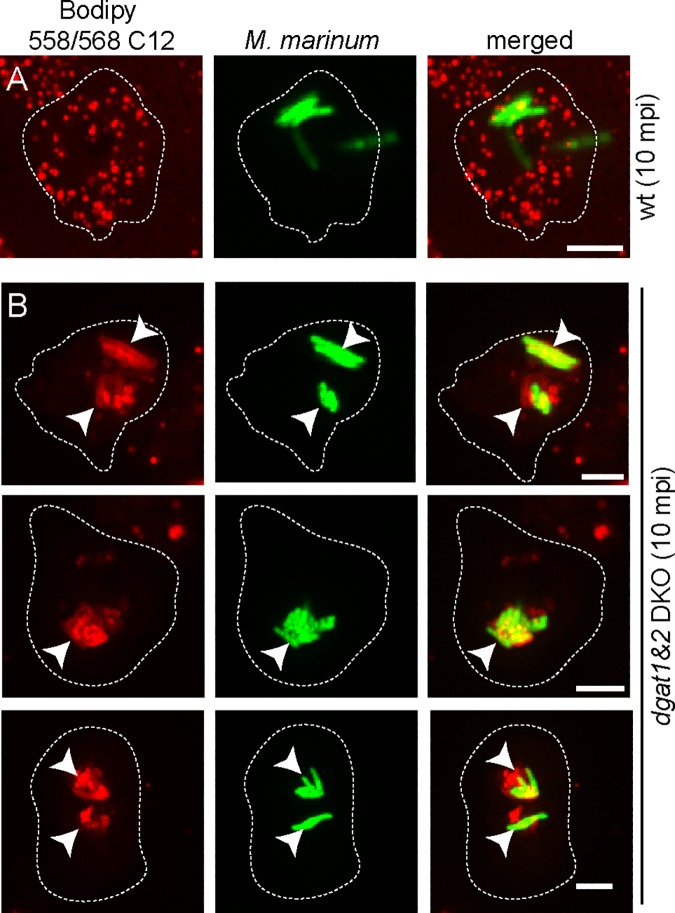
Fate of BodipyC12 –labelled host lipids early during infection of wild type and *dgat1&2* DKO. A. and B. In contrast to the wild type cells in which BodipyC12 labels host LDs (A), the fluorescent FA becomes integrated into the membrane of the MCV in *dgat1&2* DKO cells (B). *Dictyostelium* cells were labelled with BodipyC12 as described previously [12]. Wild type and *dgat1&2* DKO were infected with GFP-expressing *M*. *marinum*. Arrows point to bacteria that are in BodipyC12-labelled compartments. Images were taken 10 minutes post infection (mpi). Scale bars, 2 μm.

The situation was different when BodipyC12, an FA analogue, was used to specifically label host lipids as described previously [12]. Whereas in wild type cells, BodipyC12 becomes mainly and rapidly integrated into LDs (S4C Fig), it accumulates in the excessive ER-structures and other membranes of the *dgat1&2* DKO (S4D Fig). Interestingly, when BodipyC12-labelled *dgat1&2* DKO cells were infected, the membrane of the MCV became fluorescent minutes after phagocytosis (starting from 10 min post uptake, Fig 8B), whereas in wild type cells, the label mainly remained in LDs (Fig 8A).

**Fig 9:**
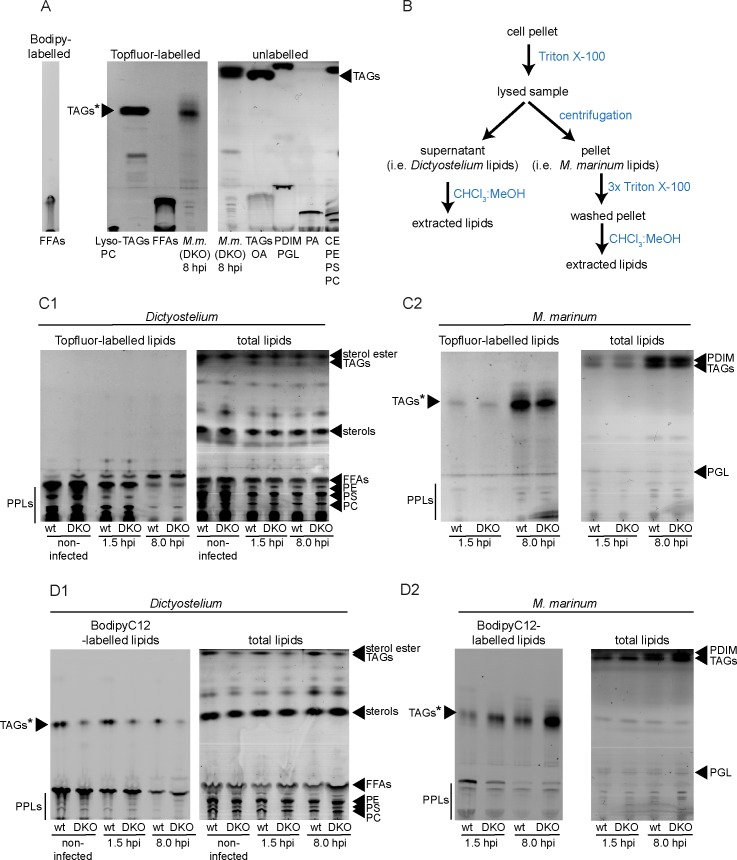
*M*. *marinum* uses host phospholipids to build up TAGs. A. Topfluor- and Bodipy-labelled lipid standards show a different migration behaviour than unlabelled standards. The migration of BodipyC12, Topfluor-FFAs (C11), Topfluor-TAGs (TAGs*; 18:1, 18:1, C11) and Topfluor-LysoPC was compared to unlabelled standards for TAGs (Triolein) and FAs (oleic acid (OA)). Additionally, the migration of PDIM, phenolic glycolipids (PGL), phosphatidic acid (PA), cholesterol esters (CE), PE, PS and PC was monitored by using the respective standards. B. Scheme showing how lipids of host and pathogen were separated prior to extraction with chloroform/methanol. C. and D. *M*. *marinum* incorporates host-derived lipids into TAGs. Wild type and *dgat1&2* DKO cells were labelled with Topfluor-LysoPC (C) and BodipyC12 (D) as described in materials and methods. Cells were infected with unlabelled *M*. *marinum*. At the indicated time points samples were taken for lipid extraction. To separate host (C1 and D1) and bacterial lipids (C2 and D2), cells were lysed with 0.05% TritonX-100 and the lipids of the pellet (bacterial lipids) were extracted with chloroform:methanol (1:2) for 24 hrs. The lipids of the supernatant (host lipids) were directly extracted with chloroform:methanol (1:2). Bands were identified by comparison with lipid standards. PPLs: phospholipids.

Another way to quantify various lipid species and to monitor a possible relocation of lipids from the host to the pathogen is via thin-layer chromatography (TLC) [7]. Again, we used Topfluor-LysoPC and BodipyC12 to follow lipid transfer from the host to the pathogen. Because of the Topfluor and Bodipy fluorophores, we expected a different migration behaviour of the tagged lipids compared to their unlabelled counterparts. Therefore, we assessed the migration of BodipyC12, Topfluor-FAs (C11), Topfluor-TAGs (18:1, 18:1, C11) and of the unlabelled lipid standards side-by-side (Fig 9A). In contrast to free FAs (FFAs, oleic acid vs. Topfluor-FAs and BodipyC12), for which no difference was observed, Topfluor-TAGs migrated less far on the TLC compared to unlabelled TAGs (Fig 9A).

To specifically label phospholipids from wild type and *dgat1&2* DKO cells, we incubated them with Topfluor-LysoPC before infection, as described above. At 1.5 and 8 hpi, samples were taken for lipid extraction. To separate the host from the pathogen, cells were gently lysed with 0.05% (v/v) TritonX-100 and centrifuged at 3,500g (Fig 9B). *Dictyostelium* lipids from the resulting supernatant were extracted directly with chloroform:methanol (1:2 v/v) whereas mycobacteria in the pellet were washed thrice with 0.05% (v/v) TritonX-100 before extraction in chloroform:methanol (1:2, v/v) for 24 hours (Fig 9B). As expected, we observed that the Topfluor-LysoPC was converted to phospholipids in wild type and *dgat1&2* DKO cells (Fig 9C1). Interestingly, the fluorescent label incorporated into phospholipids decreased during the first hours of infection and concomitantly appeared in fluorescently-labelled phospholipids and TAGs in the bacteria (Fig 9C2). When wild type and *dgat1&2* DKO cells were labelled with BodipyC12 prior to infection, we observed that the fluorescent FA analogue became mainly integrated into host phospholipids and TAGs (Fig 9D1). Fluorescently-labelled TAGs were mainly visible in the wild type, and to a smaller extent in the *dgat1&2* DKO cells. Inside the bacteria, the label was mainly incorporated into TAGs and to a smaller extent also into phospholipids (Fig 9D2). Strikingly, the bacteria were more efficient in BodipyC12 transfer and TAG synthesis in the *dgat1&2* DKO than in wild type cells (Fig 9D2).

In conclusion, we demonstrate here for the first time, that mycobacteria are not only able to obtain FAs from host TAGs, but also from host phospholipids. The phospholipids are likely hydrolysed to FAs, which are transferred into the MCV and the bacteria, to be finally used to generate TAGs and ILIs.

### *M*. *marinum* is metabolically active and stays acid-fast positive in the *dgat1&2* DKO

**Fig 10:**
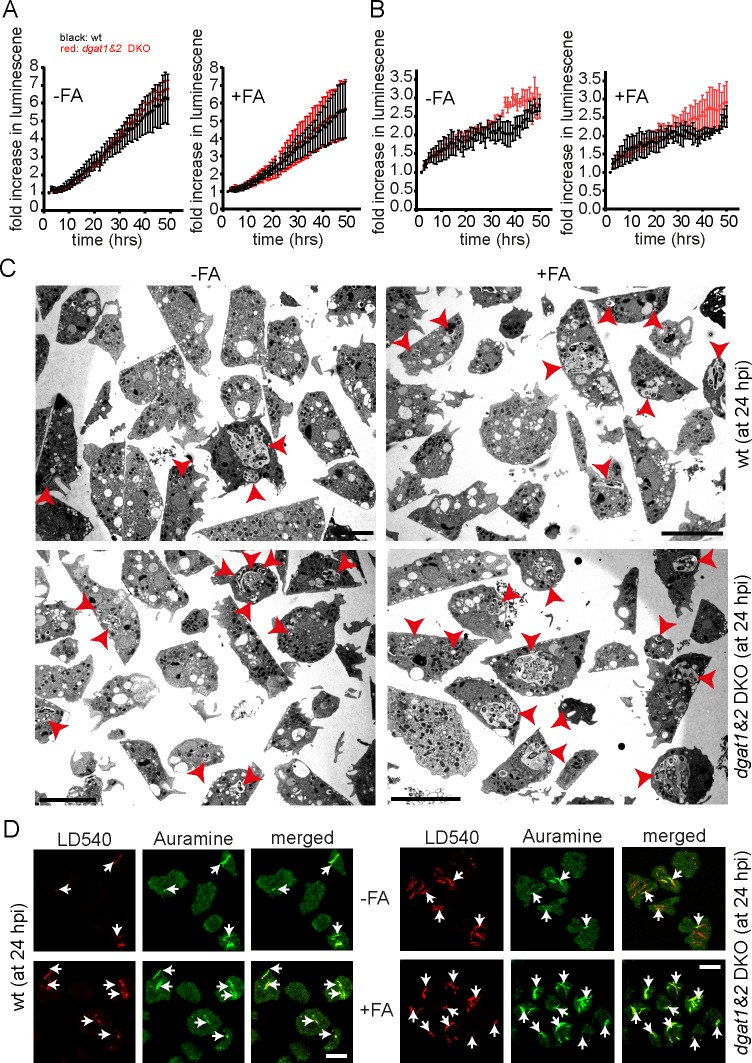
Bacteria are metabolically active and remain acid-fast positive in the *dgat1&2* DKO cells. A. Metabolic activity of *M*. *marinum* is unaltered in the *dgat1&2* DKO. Wild type and *dgat1&2* DKO cells were infected with bacteria expressing bacterial luciferase. Luminescence was recorded every hour with a microplate reader. Shown is the fold increase in luminescence over time. Symbols and error bars indicate the mean and SEM of three independent experiments. A two-way ANOVA test indicated no statistical difference between the curves. B. The number of intracellular bacteria is comparable between wild type and *dgat1&2* DKO cells. *Dictyostelium* cells were infected with mCherry-expressing *M*. *marinum*, stained with Bodipy493/503 and plated on 96-well plates. Images were recorded every hour with a high content microscope. After imaging, *Dictyostelium* cells and bacteria were segmented and analysed. Symbols and error bars indicate the mean and SEM of three independent experiments. A two-way ANOVA test indicated no statistical difference between the curves. C. *Dgat1&2* DKO cells harbour more bacteria compared to wild type cells. Wild type and the *dgat1&2* DKO cells were infected with unlabelled *M*. *marinum* wild type. At 24 hpi, cells were fixed with glutaraldehyde, stained with osmium and further processed for EM. *Dictyostelium* was fed with FAs prior to infection. Scale bars, 10 μm. D. Bacteria remain acid-fast positive in the *dgat1&2* DKO. Cells of wild type *Dictyostelium* and the *dgat1&2* DKO were infected with unlabelled *M*. *marinum*. At 24 hpi cells were fixed and subsequently stained with AuramineO and LD540. Cells were fed with FAs prior to infection where indicated. Arrows point to intracellular bacteria. Scale bars, 10 μm.

ILI accumulation has commonly been associated with the dormancy phenotype in *Mtb* [8, 31], which is accompanied by a reduction of metabolic activity and an arrest in replication. Therefore, we tested bacteria growth and metabolic activity with the help of *M*. *marinum* expressing bacterial luciferase [32]. Interestingly, no statistical difference was observed between wild type and the *dgat1&2* DKO (Fig 10A). In addition, the incubation with exogenous FAs did have neither a positive nor a negative impact on mycobacteria growth.

Overexpressing Dgat2-GFP partially complements the *dgat1* phenotype in *Dictyostelium*, leading to an increase of TAGs even without FA supplementation [19]. In this context, it is interesting that the intracellular growth of *M*. *marinum* as measured by bacterial luciferase activity was not different in wild type cells and Dgat1- or Dgat2-GFP overexpressors, even when fed with FA prior to infection (S5 Fig).

Using luciferase as a reporter measures the metabolic activity of the bacteria as a proxy for growth but does not directly inform about the actual number of intracellular and extracellular bacteria during infection. Fluorescence reporters have been validated to monitor *M*. *marinum* growth [33]. Consequently, *M*. *marinum* expressing mCherry was used to follow the infection of wild type and *dgat1&*2 DKO by high content microscopy (Figs 10B and S6). To track and segment *Dictyostelium*, we counterstained with Bodipy 493/503 (S6A Fig). Growth of *Dictyostelium* and the number of intracellular and extracellular bacteria was analysed by automatic image segmentation (Figs 10B, S6B and S6C).

Interestingly, the number of intra- and extracellular bacteria in the *dgat1&2* DKO was comparable with the wild type (Figs 10B and S6B) confirming the results above. Since the automatic segmentation did not allow to distinguish between intracellular individual bacteria and microcolonies, infected cells from wild type and *dgat1&2* DKO were fixed at 24 hpi and processed for EM. Interestingly, using this approach a higher proportion of infected cells were observed in the *dgat1&2* DKO compared to the wild type (Fig 10C). In addition, the *dgat1&2* DKO cells harboured a larger number of intracellular bacteria (arrowheads) than the control cells (Fig 10C).

In summary, we show here that *M*. *marinum* is metabolically active in the *dgat1&2* DKO and bacteria growth is comparable in wild type and *dgat1&2* DKO cells. It has been shown previously that dormant mycobacteria accumulate ILIs and lose acid-fast and/or Auramine-O staining [7, 31]. Therefore, we fixed infected wild type and *dgat1&2* DKO cells at 24 hpi and performed AuramineO and ILI-staining in parallel (Fig 10D). When *Dictyostelium* was fed with FAs prior to infection, bacteria accumulated ILIs and at the same time stayed Auramine-O-positive in both wild type and *dgat1&2* DKO cells, confirming that *M*. *marinum* does not become dormant during the infection of *Dictyostelium*.

## Discussion

**Fig 11:**
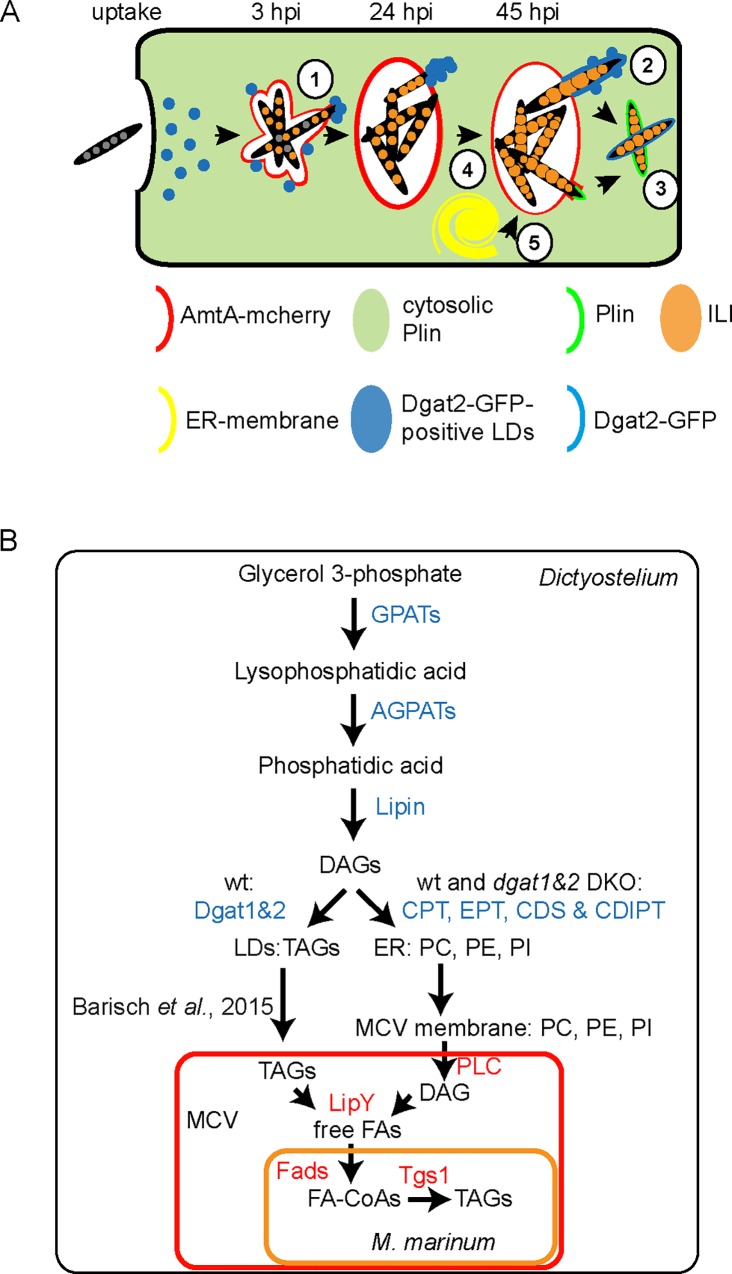
Schematic summary. A. Fate of LDs and phospholipids during infection of the *Dictyostelium* with *M*. *marinum*. **1.** Dgat2-GFP-positive LDs that are usually dispersed, cluster around bacterial poles that became exposed to the cytosol. **2.** At very late infection stages (around 45 hpi), Dgat2-GFP-positive LDs coalesce with the surface of cytosolic bacteria leading to bacteria labelling. **3.** Plin restricts Dgat2-binding to the surface of bacteria. Because Plin is recruited to LDs from a cytosolic pool, in absence of FAs it relocates faster to the cytosolic bacteria surface than Dgat2 that is always bound to LDs. **4.** Bacteria are able to accumulate numerous and large ILIs inside the *dgat1&2* DKO that is devoid of LDs. **5.** In the *dgat1&2* DKO, excess FAs is shuttled into phospholipids leading to proliferations of the ER-membrane. These phospholipids then serve as carbon source for *M*. *marinum*. B. Overview of *de novo* lipid synthesis in *Dictyostelium* wild type and *dgat1&2* DKO and lipid transfer to *M*. *marinum*. TAGs in *Dictyostelium* are mainly synthesised from glycerol-3-phosphate. The key enzymes (GPATs, AGPATs, Lipin and DGATs) are conserved between *Dictyostelium* and mammalian cells. Under normal, wild type-like conditions, TAG-filled LDs first translocate to the MCV leading to the accumulation of neutral lipids inside the compartment [12]. The delivered lipids are then used by the bacteria to build up their own TAGs and ILIs [12]. In the *dgat1&2* DKO the situation is different, since exogenous FAs are not utilized for LD biogenesis, but are shuttled into phospholipids. We propose that these phospholipids are cleaved at the membrane of the MCV by PLCs that are secreted by *M*. *marinum*. This results in the formation of DAG, which is finally hydrolysed by the secreted TAG-lipase of *M*. *marinum* (LipY (MMAR_1547)) leading to free FAs. Finally, the released free FAs are re-esterfied to bacterial TAGs probably by the activity of various Fads and Tgs1 (MMAR_1519) [13]).

In mammalian cells Dgat2 localizes to LDs, similar to other TAG synthesising enzymes probably via hairpin structures that consists of two alpha-helices ([15]; summarized in [34, 35] and [36]). Importantly, the hairpin loop does not completely span the membranes, but is embedded into the cytosolic ER leaflet and, after the re-location via ER-LD bridges, into the monolayer surrounding the LD [34, 36]. Therefore, we propose a mechanism in which Dgat2-GFP-labelled LDs first relocate to the parts of the bacteria that are exposed to the cytosol (Fig 1C and 1D; see model Fig 11A, step 1) and then sometime coalesce with the waxy bacterial cell wall (Fig 2A–2C; see model Fig 11A, step 2), thereby leading to the diffusion of Dgat2-GFP around the cytosolic bacteria. This is in line with the observation that a complete staining of the bacteria was not observed after infection with the ΔRD1 mutant, which escapes five- to ten-fold less efficiently than wild type *M*. *marinum* ([23]; Fig 2D and 2E). In analogy with our previous findings [12], we also observed LD clustering around the MCV very early during infection (Fig 4J–4L, see model Fig 11A, step 1).

In contrast to Dgat2, the *Dictyostelium* homologue of Perilipin, Plin, interacts reversibly with the surface of LDs via amphipathic helices (summarized in [36]). Plin is cytosolic when no exogenous FAs are present, i.e at later infection stages, and is therefore able to partition to the surface of the bacteria as soon as they reach the cytosol [12]. Accordingly, Plin was seen more frequently and more extensively around cytosolic bacteria than Dgat2 (S1 Fig). In conclusion, the presence of Plin might restrict Dgat2 from the surface of the bacteria.

Recently, we showed that *M*. *marinum* is able to use host LDs [12], which leads to the accumulation of ILIs. Strikingly, in *Dictyostelium* mutants deficient in one or both *dgat* genes, we observe that *M*. *marinum* was still able to build up ILIs (Figs 3 and 4A–4D, see model Fig 11A, step 4). A block in TAG synthesis, as in the *dgat1&2* DKO, does not explicitly exclude the formation of LDs. Macrophages that are deficient in both *dgat* genes, shuttle the surplus of FAs into LDs containing SEs [18]. Furthermore, yeast cells disrupted in three out of four genes relevant for the acylation of DAGs, are still capable of generating a few LDs [37]. Consequently, we tested the ability of *Dictyostelium dgat* mutants to form LDs after feeding with exogenous FAs. Interestingly, the *dgat1&2* DKO does not accumulate LDs, but shuttles excessive FAs into phospholipids, leading to massive proliferation of the ER-membrane (Fig 5 and S3 Fig). Interestingly, a similar observation was made in *S*. *cerevisiae* [38]. In yeast cells lacking all four acyltransferases, the addition of exogenous FAs leads also to massive proliferation of the ER membrane [38]. By labelling the ER-proliferations with GFP-HDEL and also by observing them by EM, we noticed that these proliferations were depleted from infected cells at very early infection stages (Fig 6) leading us to conclude that *M*. *marinum* is able to tap host phospholipids (see model Fig 11A, step 5). To monitor that the bacteria are able to access phospholipids from *Dictyostelium*, host lipids were specifically labelled with Topfluor-LysoPC, which probably became acylated giving rise to phospholipids by side-chain remodelling or head-group exchange (Fig 9C1; [39]). When Topfluor-LysoPC-labelled wild type and *dgat1&2* DKO cells were monitored by live microscopy, various vesicles were stained in the wild type, while Topfluor became visible in the ER-proliferations of the *dgat1&2* DKO (S4A and S4B Fig).

Upon infection, Topfluor-labelled lipids were first observed at the membrane of the MCV, then inside this compartment, and finally were incorporated into bacterial ILIs (Fig 7). Interestingly, a similar observation was reported during infection of HeLa cells with *Chlamydia trachomatis* [40]. The authors showed that Bodipy-PC is first enriched at the membrane of the inclusion and finally gets incorporated into the bacteria themselves [40]. By TLC, we observed that the label of host phospholipids decreased during the first hours of infection, whereas fluorescently-labelled TAGs increased in the isolated lipid fraction from the bacteria, leading to the conclusion that host phospholipids of wild type and *dgat1&2* DKO cells were successfully used as FA source (Figs 7 and 9C).

When cells were stained with the fluorescently-labelled FA analogue BodipyC12, we observed that it was incorporated into LDs in wild type cells and accumulated mainly in the proliferations of the ER-membrane in the *dgat1&2* DKO cells (S4C and S4D Fig). Therefore, we propose that BodipyC12 reveals two different routes of lipid transfer. In wild type cells, BodipyC12 is incorporated into TAGs that are transferred to the MCV in the form of LDs [12], whereas in the *dgat1&2* DKO host BodipyC12-labelled phospholipids became accessible to the bacteria (see scheme Fig 11B).

Most likely, along both routes, neither TAGs nor phospholipids are taken up by the bacteria, but both lipid species are hydrolysed to free FAs inside the MCV (see scheme Fig 11B). This hypothesis is supported by the findings from a previous study in which the authors showed that FAs released from host LDs are incorporated mainly into *Mtb* membrane lipids, such as phthiocerol dimycocerosate (PDIM) and TAGs [41]. Host TAGs in the MCV are probably hydrolysed by host lysosomal lipases or secreted bacterial lipases such as LipY [42, 43], whereas phospholipids are cleaved by host or bacterial phospholipases (PLs). PLs are classified into four major groups depending on the cleavage site. PLCs for instant hydrolyse phospholipids to generate DAG. Especially PLCs have been shown to play an important role in pathogenesis [44]. Interestingly, *Mtb* possesses four genes encoding PLCs ((plcA-D); [45]). All of them are secreted by the twin-arginine transporter (Tat) system [46] and are jointly required for virulence of *Mtb* in mice [47]. A role of PLCs in bacterial escape into the cytosol has been already demonstrated for other bacterial pathogens such as *Listeria monocytogenes* [48, 49] and *Clostridium perfringens* [50]. In contrast, the ability of *Mtb* to break its compartment was not reduced in a quadruple *plc* KO [51]. Interestingly, PLC activity seems to be restricted to slow-growing pathogenic mycobacterial species and has also been observed in *M*. *marinum* [52]. In contrast to *Mtb*, the genome of *M*. *marinum* harbours six gene duplicates of *plcB* (*plcB*_1 to *plcB*_6, http://mycobrowser.epfl.ch/ marinolist.html), but no version of *plcA*, *plcC* and *plcD*. As proposed by others [53], we suggest that mycobacterial PLCs play a role in hydrolysing host phospholipids, giving rise to DAG, making it available to the bacteria (see Fig 11B).

Because free FAs are toxic, they are either bound to proteins or are activated by coenzyme A (CoA) to yield fatty acyl-CoA. Interestingly, *Mtb* has 36 FadD genes (38 in the case of *M*. *marinum*) annotated as putative fatty acyl-CoA synthase genes [45]. Although some of them have been identified as fatty acyl-AMP ligases that are involved in lipid synthesis such as FAdD32 [54], others might catalyse the activation of FAs with Coenzyme A. In addition, FACL6 (Rv1206), another putative fatty acyl-CoA synthase that resembles FA transport proteins of mammalian cells, displays preference towards oleic acid, one of the predominant host FAs, and is upregulated during *in vitro* dormancy [55].

In summary, we propose here a new mechanism by which mycobacteria exploit lipids from their host cell. In addition to host TAGs which are transferred to the MCV by LD relocation and translocation [12], the bacteria are also able to manipulate and access host phospholipids that are cleaved and probably transported into the bacteria in the form of free FAs. FAs released from host TAGs [12] and phospholipids become integrated into bacterial ILIs in wild type hosts and to an even higher extent in the *dgat1&2* DKO. Since ILI formation is a major characteristic of dormant bacteria, we monitored mycobacteria growth by high content microscopy and metabolic activity using bacterial luciferase (Fig 10A and 10B). Using these methods we did neither observe a reduction in metabolic activity nor in bacteria growth. Interestingly, more infected cells and an increase in the number of intracellular bacteria became visible in the *dgat1&2* DKO in EM-micrographs (Fig 10C). In addition, the bacteria remained acid-fast positive, leading us to the conclusion that a dormancy-like phenotype was not induced in *Dictyostelium* under these conditions (Fig 10D).

## Materials and Methods

### Dictyostelium plasmids, strains and cell culture

All the *Dictyostelium* material used for this article is listed below (S1 Table). Wild type *Dictyostelium* was grown axenically at 22°C in Hl5c medium (Foremedium). To avoid bacterial growth, the medium was supplemented with 100 U/ml of penicillin and 100 μg/ml streptomycin (Invitrogen). To monitor the localization of Dgat1 and Dgat2 during infection, plasmids carrying Dgat1-GFP (#752) and Dgat2-GFP (#622, both [19]) were transformed into AX2 wild type. The bacterium-containing compartment in Dgat2-GFP-expressing or *dgat1&2* DKO cells, respectively, was labelled with AmtA-mCherry as previously described [12]. In addition, Dgat2-GFP-expressing cells were transformed with a RFP-Plin-carrying plasmid [12]. To have a ER-marker for live imaging, the *dgat1&2* DKO cells were transformed with a plasmid harbouring GFP-HDEL [28]. After electroporation, selection with hygromycin (50 μg/ml), neomycin (5 μg/ml) and blasticidin (5 μg/ml) was applied. For the viability test, propidium iodide (PI) was purchased from Fluka and digitonin from Sigma.

The *dgat* knockout cells lines were generously provided by Prof. Maniak [19].

### Mycobacteria plasmids, strains and culture

*M*. *marinum* was cultured in shaking conditions at 150 rpm in 7H9 medium (Becton Dickinson) supplemented with 10% OADC, 0.05% Tween80 and 0.2% glycerol at 32°C. To minimize clumping of the bacteria, Erlenmeyer flasks containing 5 mm glass beads were used. Cultures that were used for infection were grown until OD_600_ of 1 (1.5 x 10^8^ bacteria/ml). The *M*. *marinum* M strain was generously provided by L. Ramakrishnan (Washington University). *M*. *marinum* expressing bacterial luciferase and the ΔRD1 mutant were generated in the Soldati laboratory [32] and were grown in the presence of 25 μg/ml kanamycin. Bacteria expressing the pCherry10 plasmid [56] were grown in medium supplemented with 100 μg/ml hygromycin. The *M*. *marinum* strain expressing GFP [57] was cultivated in the presence of 25 μg/ml kanamycin.

### Infection of *Dictyostelium* with *M. marinum*

To monitor events that happen directly after phagocytosis of *M*. *marinum*, an early infection assay was performed as previously described [58]. Briefly, 5 x 10^8^ bacteria were centrifuged at 18,000 g for 4 min and washed twice with filtered Hl5c. Before the infection, the bacteria were passaged 10 times through a 25-gauge needle to remove clumps. Forty microliters of bacterial suspension (multiplicity of infection (MOI) of 2) were used to infect *Dictyostelium* cells on a confluent μ-dish (ibidi) in low fluorescent medium (LoFlo, Foremedium) without antibiotics. To increase synchronous uptake of *M*. *marinum*, the μ-dish was cooled for 10 min on a cold metal plate. The bacteria were centrifuged on the *Dictyostelium* cells for 2 min at 500 g and 4°C. Where indicated, the medium was exchanged to medium containing 10 μM Bodipy 493/503 (stock: 10 mM in DMSO) to stain neutral lipids. To improve imaging quality the infection was overlayed with a thin sheet of agarose as previously described [58]. Time-laps movies or stacked images were recorded at a spinning disc confocal microscope (Intelligent Imaging Innovations) mounted on an inverted microscope (Leica DMIRE2; Leica) using the 63x NA or 100x 1.4 NA oil objective and an Evolve EMCCD Camera (Photometrics). Images were processed afterwards by using FIJI.

To monitor the whole infection time course between 2 and 60 hpi, a long-term infection experiment, was performed as previously described ([22]; [58]). 5 x 10^8^ bacteria (for a final MOI of 10) were washed twice with filtered Hl5c and resuspended in 500 μl Hl5c. To remove clumps, bacteria were passaged 10 times though a 25-gauge needle and added to a confluent dish of *Dictyostelium* cells in filtered Hl5c that have been incubated without antibiotics for 24 hrs. The bacteria were centrifuged onto the adherent *Dictyostelium* cells at 500 g and 25°C for 2 x 10 min to increase phagocytosis efficiency. After an additional 10 to 20 min incubation, the extracellular bacteria were removed by several washes in filtered Hl5c. Finally, the infection was taken up in 30 ml filtered Hl5c to have a density of 1 x 10^6^ cells/ml and supplemented with 5 μg/ml of streptomycin and 5 U/ml of penicillin to prevent growth of extracellular bacteria and incubated in shaking conditions at 25°C at 130 rpm. At the indicated time points, samples were taken for PFA/picric acid fixation or live imaging as previously described [12].

To induce LDs in *Dictyostelium* prior to infection, the axenic growth medium was supplemented with 200 μM palmitate. After 3 hrs of incubation and before the bacteria were added, exogenous FAs were removed by several washing steps. To prepare a 0.1 mM stock solution, sodium palmitate was dissolved in pre-warmed MeOH. Aliquots were stored at -20°C.

Bacterial growth was assessed by high content live microscopy (ImageXpress Micro XL, Molecular Devices). 1 x 10^5^ *Dictyostelium* cells that were infected either with GFP- or mCherry-expressing *M*. *marinum* were plated on a multi-well imaging plate (BD Falcon) and images were recorded every hour using a 40 x air objective. Images were processed using automated segmentation and quantitation with the help of the software MetaXpress (Molecular Devices). Bodipy 493/503 was used where indicated.

Metabolic growth was measured with the help of *M*. *marinum* expressing bacterial luciferase as previously described [32]. Briefly, infected *Dictyostelium* cells (dilutions between 0.5 and 2.0 × 10^5^) were plated on non-treated, white F96 MicroWell™ plates (Nunc) and covered with a gas permeable moisture barrier seal (Bioconcept). Luminescence was measured at a constant temperature of 25°C every hour for around 50 hours using a Synergy Mx Monochromator Based Multi-Mode Microplate Reader (Biotek).

### Antibodies, fluorescent dyes and immunofluorescence

Bodipy 493/503, Bodipy 558/568 (BodipyC12) and Vbyrant Ruby were purchased from Thermo Scientific. LD540 was a gift from Prof. Christoph Thiele (Bonn, Germany). The AuramineO staining was performed using the TB Fluorescent Stain Kit M (Becton Dickinson). The p80-antibody [59] was obtained from the lab of Dr. Pierre Cosson (University of Geneva). Goat-anti mouse IgG coupled to Alexa488 or Alexa647 (Thermo Scientific) were used as secondary antibodies. Topfluor-LysoPC, Topfluor-FAs (C11) and Topfluor-TAGs (18:1, 18:1, C11) were purchased from Avanti Polar Lipids.

*Dictyostelium* was fixed with 4% PFA/picric acid or ultra cold methanol as described previously [60]. Staining with Bodipy 493/503 at a final concentration of 20 μM was performed in parallel with the secondary antibody. Images were recorded with a Zeiss LSM700 confocal microscope using a 63× 1.4 NA or 100× 1.4 NA oil immersion objective.

### Electron microscopy

A confluent 6-cm dish of infected *Dictyostelium* was fixed for one hour with 2% glutaraldehyde in Hl5c and stained for 20 min with imidazole (0.1 M)-buffered 2% osmium tetroxide, as previously described ([61]; [12]). Afterwards cells were collected using a cell scraper and resuspended in 1 ml of phosphate-buffered saline (PBS) in an Eppendorf tube. After two washes with PBS, the samples were sent to the EM platform of the Faculty of Medicine, University of Geneva for further processing. There, samples were postfixed, embedded in Epon resin and further processed as previously described [62]. Images were taken with a Tecnai 20 electron microscope (FEI).

### Pulse chase with fluorescent lipids and TLC

*Dictyostelium* was incubated overnight with exogenous FAs to induce LDs in the wild type or ER-membrane proliferations in the *dgat1&2* DKO. Topfluor-LysoPC (final concentration: 1 μM, stock: 3 mM in methanol) was added complexed to defatted BSA in a molecular ratio of 2:1. Before addition, exogenous FAs were removed by three washes with sterile filtered Hl5c at 500 g for 5 min. After one hour incubation with Topfluor-LysoPC, non-metabolized dye was removed by three washes and a one hour chase to avoid labelling of extracellular bacteria.

*Dictyostelium* was incubated overnight with exogenous FAs and at the same time with Bodipy 558/568 C12 (final concentration: 5 μM, stock: 2.1 mM in DMSO) to induce LDs in the wild type or ER-membrane proliferations in the *dgat1&2* DKO cells. To remove non-metabolized dye, cells were washed three times and chased for 1 hour in sterile filtered Hl5c.

For live imaging, an early infection experiment was performed as described previously [58]. Therefore, cells were allowed to adhere on a μ-dish (ibidi) and subsequently infected with *M*. *marinum* expressing mCherry or GFP. At the indicated time points, images were taken with a spinning disc confocal microscope.

To follow lipid transfer from the host to the pathogen by TLC, 4 confluent 10-cm dishes of Topfluor-LysoPC- or Bodipy558/568 C12-labelled wild type and *dgat1&2* DKO cells were infected with unlabelled *M*. *marinum* wild type. At 1.5 and 8 hpi, two dishes of each strain (7x10^7^ cells) were collected at 500 g for 5 min. After two washes with Soerensen buffer, cell pellets were snap frozen in liquid nitrogen and stored at– 20°C until lipids were extracted. Two confluent 10-cm dishes with non-infected cells served as a control and were treated like the infected cells.

First, *Dictyostelium* cells were lysed in water containing 0.05% Triton X-100 (v/v), sonicated and centrifuged at 3500g. The pellet containing *M*. *marinum* was washed thrice with 0.05% TritonX-100 before a 24 hour lipid extraction was performed with chloroform:methanol (ratio 1:2 (v/v)). *Dictyostelium* lipids were isolated from the 3500 x g supernatant by chloroform:methanol extraction (ratio 1:2 (v/v); [63]).

After the extraction, lipid samples were spotted on silica TLC plates (Merck Millipore). Phospholipids were separated in a system containing chloroform/ethanol/water/triethylamine 35:40:9:35 [64]. After one third of the total separation distance, the plates were dried and subsequently placed in a system containing isohexane/ethylacetate 5:1 to separate neutral lipids [64]. Images from fluorescent lipids were taken under UV illumination (312 nm). FIJI was used to invert the images and to convert to gray scale. Total lipids were detected by charring with a solution containing magnesium chloride, methanol and sulfuric acid. Lipid standards were obtained from Avanti Polar Lipids and Biodefense and Emerging Infections Research (BEI) resources (NIAID, NIH) and used to identify bands.

## Supporting Information

S1 Fig Perilipin restricts Dgat2 from the surface of cytosolic bacteria. A. Bacteria that are heavily decorated with RFP-Plin are less labelled by Dgat2-GFP and vice versa. Yellow arrows point to bacteria that are positive for RFP-Plin, white arrows label bacteria that are stained with Dgat2-GFP. Scale bars, 5 μm. B. Bacteria are decorated more frequently with RFP-Plin than with Dgat2-GFP. *Dictyostelium* cells expressing RFP-Plin and Dgat2-GFP were infected with unlabelled *M*. *marinum*. Samples were taken at the indicated time points and *M*. *marinum* stained with Vybrant Ruby. Maximum z-projections were analysed for *Dictyostelium* cells that harboured RFP-Plin or Dgat2-GFP-positive bacteria. The statistical significance was calculated with an unpaired t-test (* p<0.05, ** p<0.01). Bars represent the mean and SD of two independent experiments.
(TIF)

S2 Fig Viability of the *dgat1&*2 DKO is unchanged in cells treated with FAs. The viability of wild type and *dgat1&*2 DKO is unchanged after incubation with FAs (A). As expected both cell lines are susceptible to digitonin (B). Images were taken three hours after incubation with FAs or digitonin. The viability was monitored using PI. Arrows point to dead cells that are positive for PI. Scale bars, 10 μm.
(TIF)

S3 Fig LDs are formed in wild type and *dgat2* KO cells, but not in the *dgat1* KO and the *dgat1&2* DKO. While wild type (A) and *dgat2* KO cells (C) produce LDs after 3 hrs feeding with FAs, long ER-strands (red arrows) are observed in the *dgat1* single KO (B). Instead of LDs, ER-membranes proliferations became visible in the *dgat1&*2 DKO (D). Red asterisks label mitochondria that are close to the ER-membrane proliferations. LDs are labelled with blue arrowheads. Scale bars, 2 μm. E. In cells expressing GFP-HDEL, BodipyC12 becomes incorporated into ER-membrane proliferations after incubation with FAs. Scale bar, 5 μm, Zoom 2 μm.
(TIF)

S4 Fig Topfluor-LysoPC and BodipyC12 labelling in non-infected cells after feeding with FAs. A. and B. Topfluor-LysoPC is enriched in vesicles in the wild type (A) and in ER-membrane proliferations in the *dgat1&2* DKO (B). Asterisks label the nuclei. C. and D. Bodipy 558/568 C12 is mainly enriched in LDs in the wild type (C) and in ER-membrane proliferations in the *dgat1&2* DKO cells (D). *Dictyostelium* was stained with Topfluor-LysoPC, BodipyC12 and Bodipy493/503 as described in materials and methods. Arrowheads point to ER-proliferations. Scale bars 10 μm, Zoom 2 μm.
(TIF)

S5 Fig Bacteria growth in Dgat1-GFP- and Dgat2-GFP-expressing *Dictyostelium*. Bacterial growth is unaltered in cells overexpressing Dgat1 and Dgat2. Wild type and Dgat1- and Dgat2-GFP-expressing cells were infected with *M*. *marinum* expressing bacterial luciferase. Luminescence was recored every hour with a microplate reader. Shown is the fold increase in luminescence over time. Symbols and error bars indicate the mean and SEM of three independent experiments.
(TIF)

S6 Fig *M*. *marinum* growth is unaltered in *dgat1&2* DKO cells. A. *Dictyostelium* cells and *M*. *marinum* bacteria are well detected by MetaXpress. Yellow: non-infected *Dictyostelium*, blue: infected *Dictyostelium*, red: extracellular bacteria, white: intracellular bacteria. Shown are images taken at 50 hpi. B. The number of extracellular bacteria assessed by high-content microscopy. C. *Dictyostelium* growth assessed by high-content microscopy. *Dictyostelium* cells were infected with mCherry-expressing bacteria, stained with Bodipy493/503 and plated on a 96-well plates. Images were recorded every hour with a high content microscope. After imaging, *Dictyostelium* cells and bacteria were segmented with MetaXpress (Molecular Devices). Symbols and error bars indicate the mean and SEM of three independent experiments. Statistical differences were calculated with a Bonferroni post hoc test after two-way ANOVA. Significantly different values were indicated by an asterisk (**P < 0.01, ***P < 0.001).
(TIF)

S1 Movie RFP-Plin and Dgat2-GFP dynamics after feeding with exogenous FAs. For more information, see Fig 1B.
(MP4)

S2 Movie LDs are attached to the bacterial surface and co-ordinately move along with the bacteria. For more information, see Fig 2B.
(MP4)

S3 Movie One LD coalesces with the bacterial surface. For more information, see Fig 2C.
(MP4)

S4 Movie Bodipy 493/503 staining of wild type and various *dgat* mutants after feeding with exogenous FAs. For more information, see Fig 5A.
(MP4)

S1 Table *Dictyostelium* material used for this publication. (DOCX)
