# Supplementary figures and images for: Mycobacterium marinum Degrades Both Triacylglycerols and Phospholipids from Its Dictyostelium Host to Synthesise Its Own Triacylglycerols and Generate Lipid Inclusions
Source: PLoS Pathog. 2017 Jan 19;13(1):e1006095. doi: 10.1371/journal.ppat.1006095 (PMC5245797; doi:10.1371/journal.ppat.1006095)

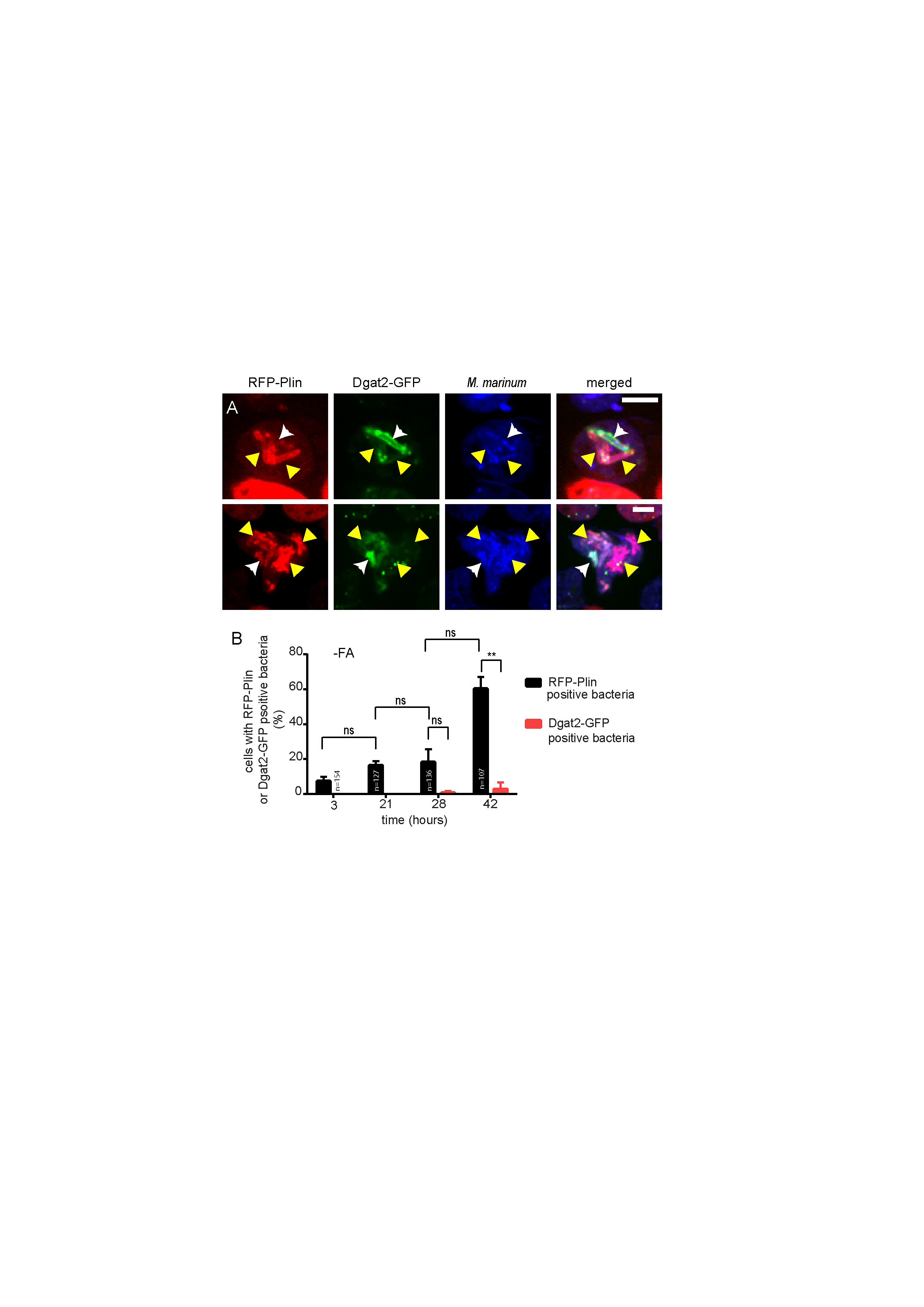

Supplement: S1 Fig — A. Bacteria that are heavily decorated with RFP-Plin are less labelled by Dgat2-GFP and vice versa. Yellow arrows point to bacteria that are positive for RFP-Plin, white arrows label bacteria that are stained with Dgat2-GFP. Scale bars, 5 μm. B. Bacteria are decorated more frequently with RFP-Plin than with Dgat2-GFP. Dictyostelium cells expressing RFP-Plin and Dgat2-GFP were infected with unlabelled M. marinum. Samples were taken at the indicated time points and M. marinum stained with Vybrant Ruby. Maximum z-projections were analysed for Dictyostelium cells that harboured RFP-Plin or Dgat2-GFP-positive bacteria. The statistical significance was calculated with an unpaired t-test (* p<0.05, ** p<0.01). Bars represent the mean and SD of two independent experiments. (TIF) [file ppat.1006095.s001.tif]

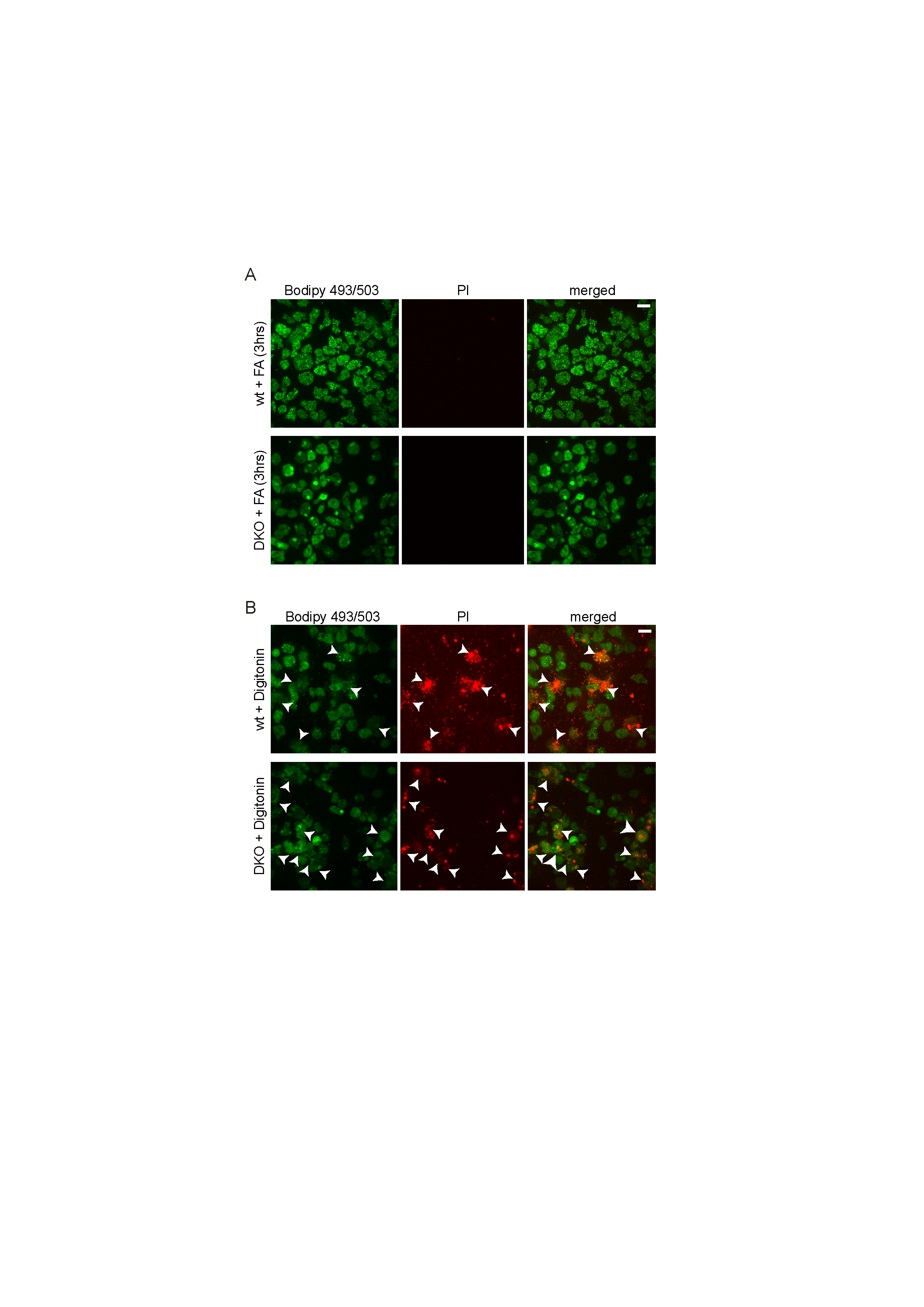

Supplement: S2 Fig — The viability of wild type and dgat1&2 DKO is unchanged after incubation with FAs (A). As expected both cell lines are susceptible to digitonin (B). Images were taken three hours after incubation with FAs or digitonin. The viability was monitored using PI. Arrows point to dead cells that are positive for PI. Scale bars, 10 μm. (TIF) [file ppat.1006095.s002.tif]

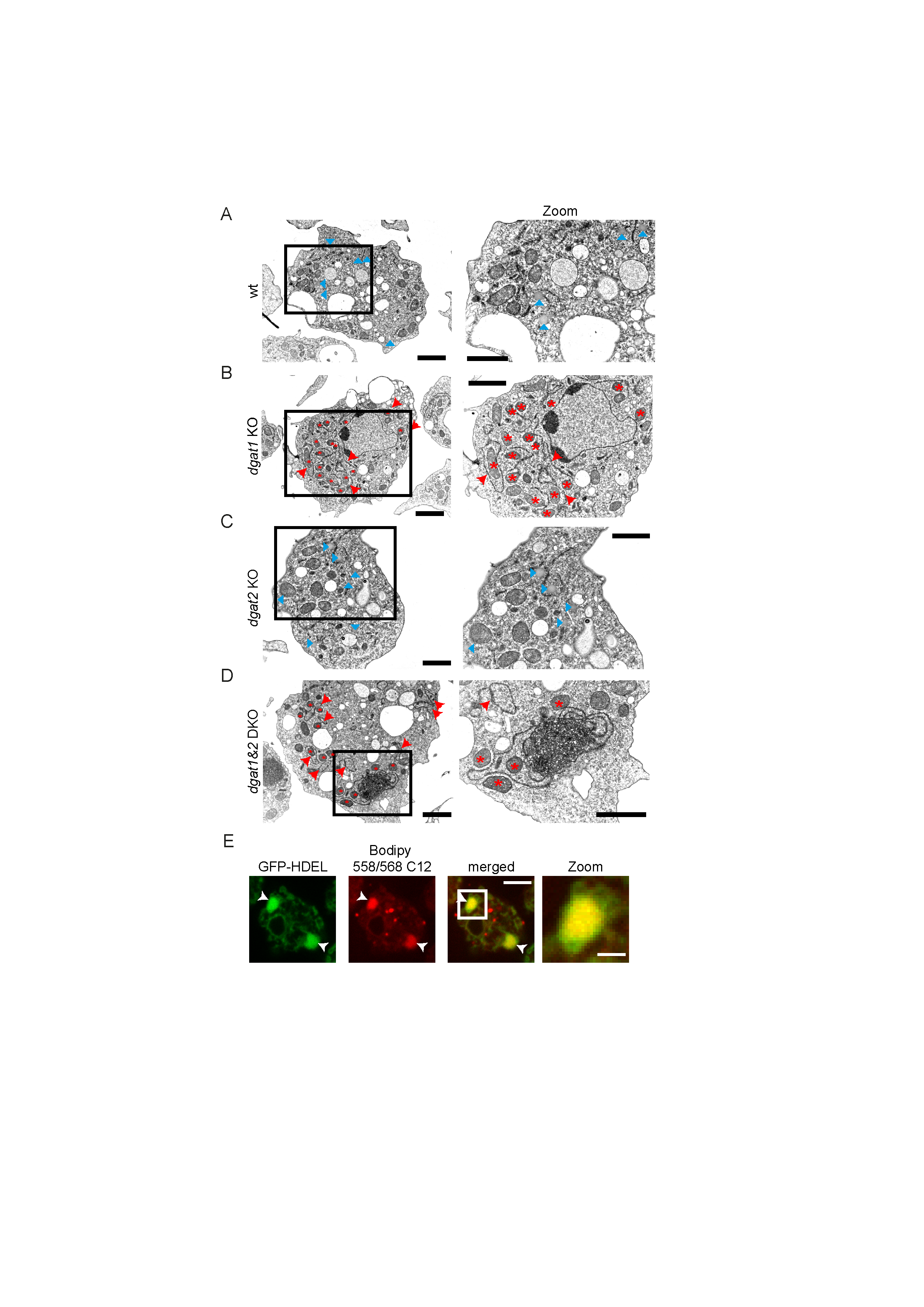

Supplement: S3 Fig — While wild type (A) and dgat2 KO cells (C) produce LDs after 3 hrs feeding with FAs, long ER-strands (red arrows) are observed in the dgat1 single KO (B). Instead of LDs, ER-membranes proliferations became visible in the dgat1&2 DKO (D). Red asterisks label mitochondria that are close to the ER-membrane proliferations. LDs are labelled with blue arrowheads. Scale bars, 2 μm. E. In cells expressing GFP-HDEL, BodipyC12 becomes incorporated into ER-membrane proliferations after incubation with FAs. Scale bar, 5 μm, Zoom 2 μm. (TIF) [file ppat.1006095.s003.tif]

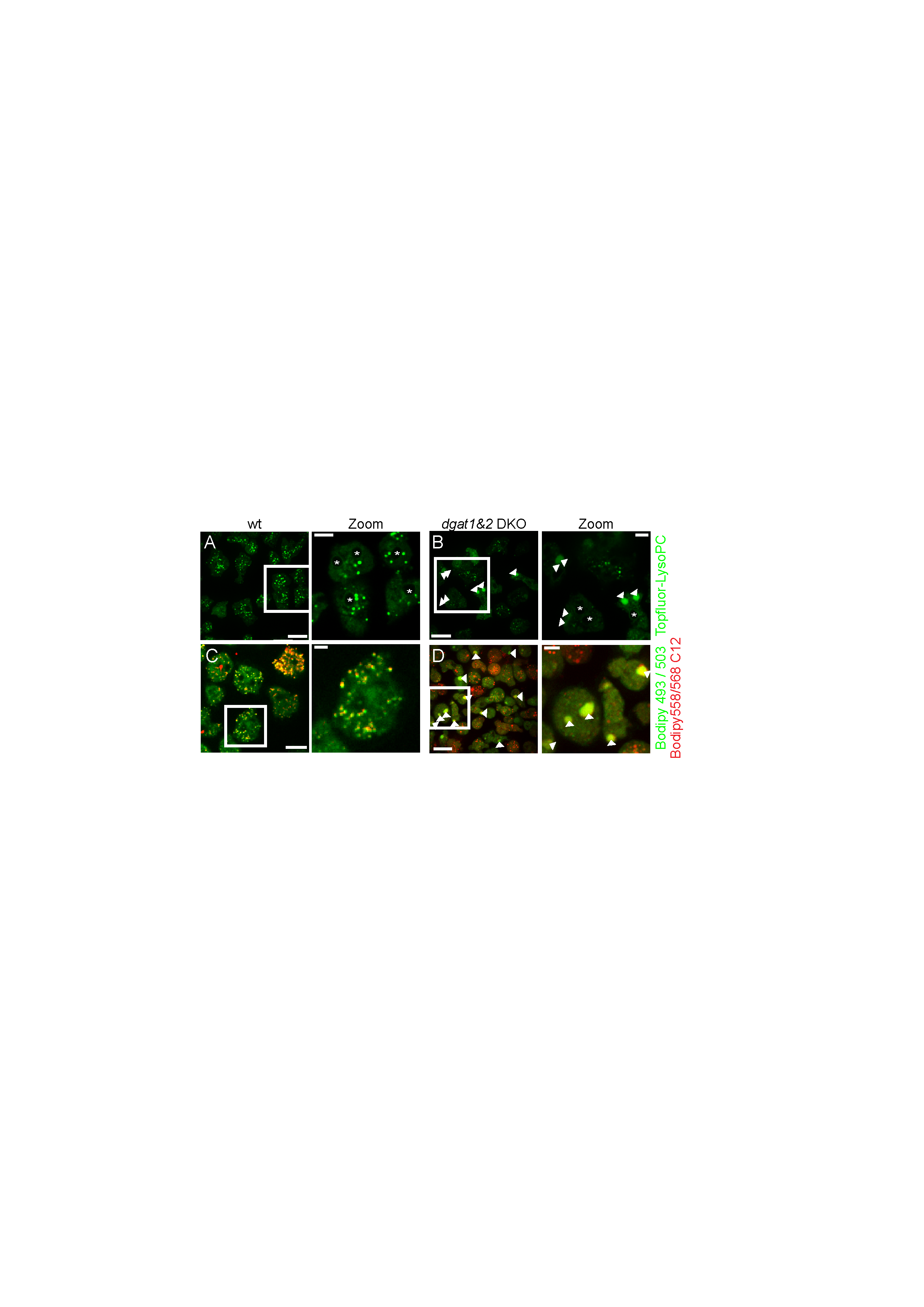

Supplement: S4 Fig — A. and B. Topfluor-LysoPC is enriched in vesicles in the wild type (A) and in ER-membrane proliferations in the dgat1&2 DKO (B). Asterisks label the nuclei. C. and D. Bodipy 558/568 C12 is mainly enriched in LDs in the wild type (C) and in ER-membrane proliferations in the dgat1&2 DKO cells (D). Dictyostelium was stained with Topfluor-LysoPC, BodipyC12 and Bodipy493/503 as described in materials and methods. Arrowheads point to ER-proliferations. Scale bars 10 μm, Zoom 2 μm. (TIF) [file ppat.1006095.s004.tif]

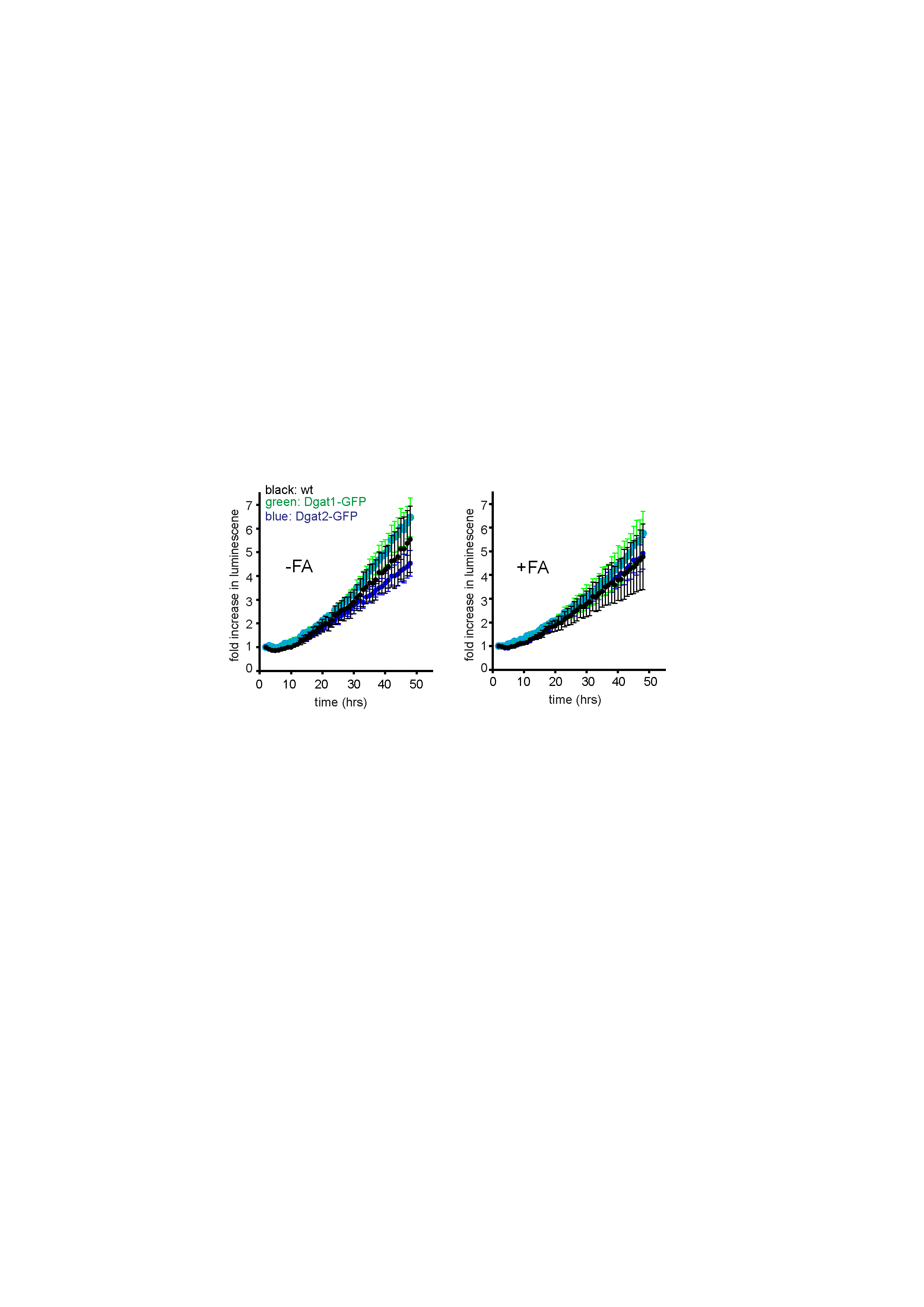

Supplement: S5 Fig — Bacterial growth is unaltered in cells overexpressing Dgat1 and Dgat2. Wild type and Dgat1- and Dgat2-GFP-expressing cells were infected with M. marinum expressing bacterial luciferase. Luminescence was recored every hour with a microplate reader. Shown is the fold increase in luminescence over time. Symbols and error bars indicate the mean and SEM of three independent experiments. (TIF) [file ppat.1006095.s005.tif]

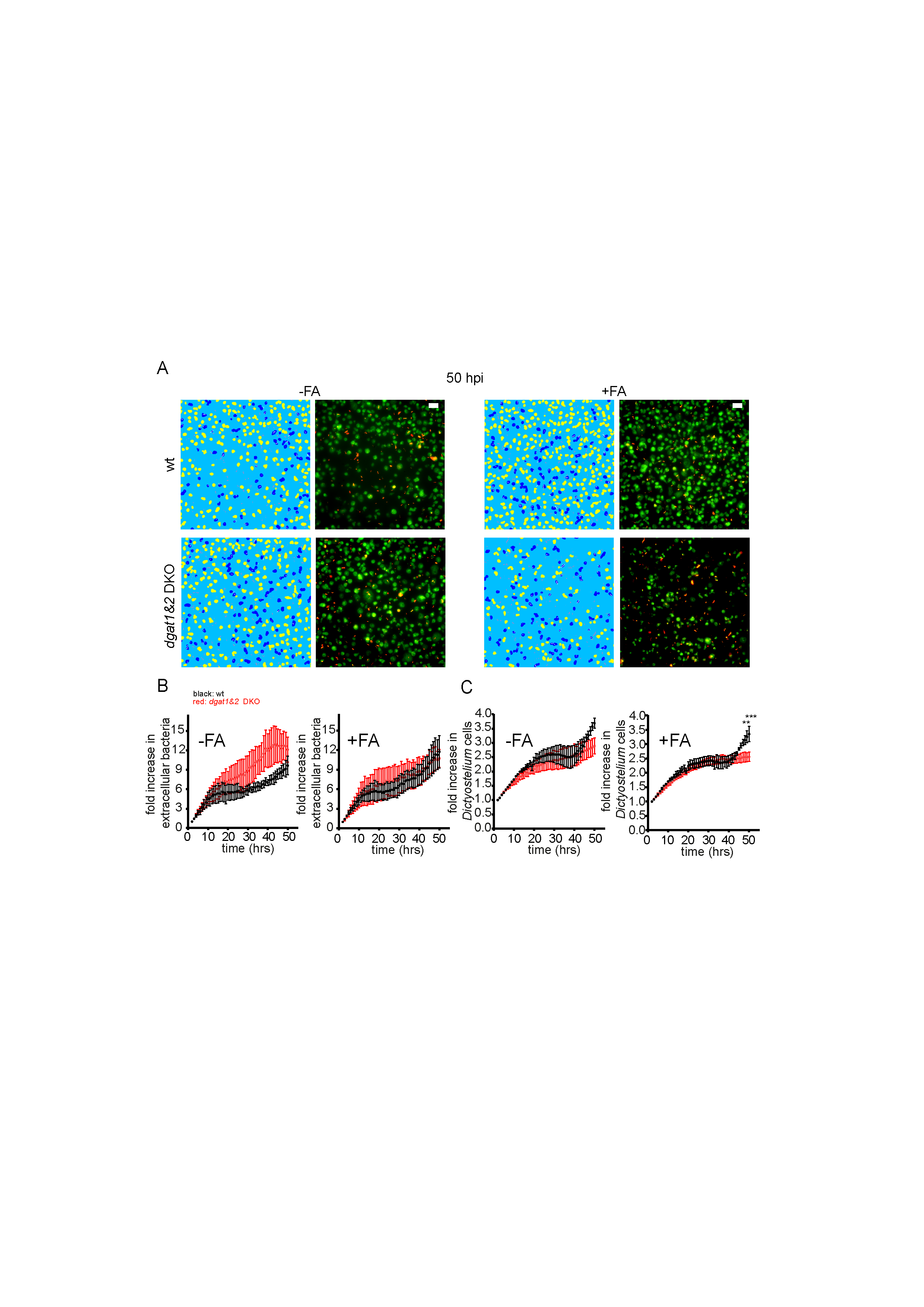

Supplement: S6 Fig — A. Dictyostelium cells and M. marinum bacteria are well detected by MetaXpress. Yellow: non-infected Dictyostelium, blue: infected Dictyostelium, red: extracellular bacteria, white: intracellular bacteria. Shown are images taken at 50 hpi. B. The number of extracellular bacteria assessed by high-content microscopy. C. Dictyostelium growth assessed by high-content microscopy. Dictyostelium cells were infected with mCherry-expressing bacteria, stained with Bodipy493/503 and plated on a 96-well plates. Images were recorded every hour with a high content microscope. After imaging, Dictyostelium cells and bacteria were segmented with MetaXpress (Molecular Devices). Symbols and error bars indicate the mean and SEM of three independent experiments. Statistical differences were calculated with a Bonferroni post hoc test after two-way ANOVA. Significantly different values were indicated by an asterisk (**P < 0.01, ***P < 0.001). (TIF) [file ppat.1006095.s006.tif]
