# Supplementary material for: Mycobacterium marinum Degrades Both Triacylglycerols and Phospholipids from Its Dictyostelium Host to Synthesise Its Own Triacylglycerols and Generate Lipid Inclusions
Source: PLoS Pathog. 2017 Jan 19;13(1):e1006095. doi: 10.1371/journal.ppat.1006095 (PMC5245797; doi:10.1371/journal.ppat.1006095)
Supplement: S1 Table — (DOCX) [file ppat.1006095.s011.docx]

**S1 Table. *Dictyostelium* material used for this study.**

| ***Dictyostelium* strains** | **Plasmids used for transformation** | **Reference** |
| --- | --- | --- |
| AX2 (wild type) |  |  |
| Dgat1-GFP | pDd-A15-Dgat1-GFP (#752) | [[19](#_ENREF_19)] |
| Dgat2-GFP | pDd-A15-Dgat2-GFP (#622) | [[19](#_ENREF_19)] |
| Dgat2-GFP/  AmtA-mCherry | see above for Dgat2-GFP/  pDM1044-AmtA-mCherry | [[12](#_ENREF_12)] |
| Dgat2-GFP/  mRFPmars-Plin | see above for Dgat2-GFP/  pDEXRH-mRFPmars-Plin | [[12](#_ENREF_12)] |
| *dgat1* KO | pGEM-T Easy-*dgat1*-BS^r^ (pLPBLP) | [[19](#_ENREF_19)] |
| *dgat2* KO | pGEM-T Easy-*dgat2*-BS^r^ (pLPBLP) | [[19](#_ENREF_19)] |
| *dgat1&2* DKO | *dgat1* KO 1-17, after removal of the BS^r^  cassette by pDexRHNLS-Cre, transformation with pGEM-T Easy-*dgat2*-BS^r^ (pLPBLP) | [[19](#_ENREF_19)] |
| *dgat1&2* DKO/  AmtA-mCherry | See above for the *dgat1&2* DKO/  pDM1044-AmtA-mCherry | [[12](#_ENREF_12)] |
| GFP-HDEL | pAC6-GFP-HDEL | [[28](#_ENREF_28)] |
| *dgat1&2* DKO/  GFP-HDEL | see above | [[28](#_ENREF_28)] |
